# Solvent Effect on the Structural, Optical, Morphology, and Antimicrobial Activity of Silver Phosphate Microcrystals by Conventional Hydrothermal Method

**DOI:** 10.1021/acsomega.4c02943

**Published:** 2024-05-16

**Authors:** Mitsuo Lopes Takeno, Francisco Xavier Nobre, Fagner Ferreira da Costa, Marcus Valério Botelho do Nascimento, Wanison André Gil Pessoa Júnior, Edgar Alves Araújo Júnior, Giancarlo da Silva Sousa, Marcel Leiner de Sá, Raiana Silveira Gurgel, Patrícia Melchionna Albuquerque, José Milton Elias de Matos, Yurimiler Leyet Ruiz, Carlos Roberto Grandini

**Affiliations:** # Department of Chemistry, Environment, and Food (DQA), Group of Energy Resources and Nanomaterials (GREEN), Federal Institute of Education, Science and Technology of Amazonas, Campus Manaus Centro, Manaus, 69020-120, AM Brazil; ‡ Interdisciplinar Laboratory of Advanced Materials-LIMAV, Federal University of Piauí-UFPI, Teresina, 64049-550 PI Brazil; § Research Group on Chemistry Applied to Technology, School of Technology, Amazonas State University, Manaus 69050-020, Brazil; ∥ Department of Materials Engineering, Laboratory of Processing of Technological Materials (LPMaT), Federal University of Amazonas, Faculty of Technology, Rua Av. General Rodrigo Otávio Jordão Ramos, 1200, Coroado I, Manaus, 69067-005, Brazil; ⊥ Laboratório de Anelasticidade e Biomateriais, UNESP−Universidade Estadual Paulista, Bauru 17033-360, SP Brazil

## Abstract

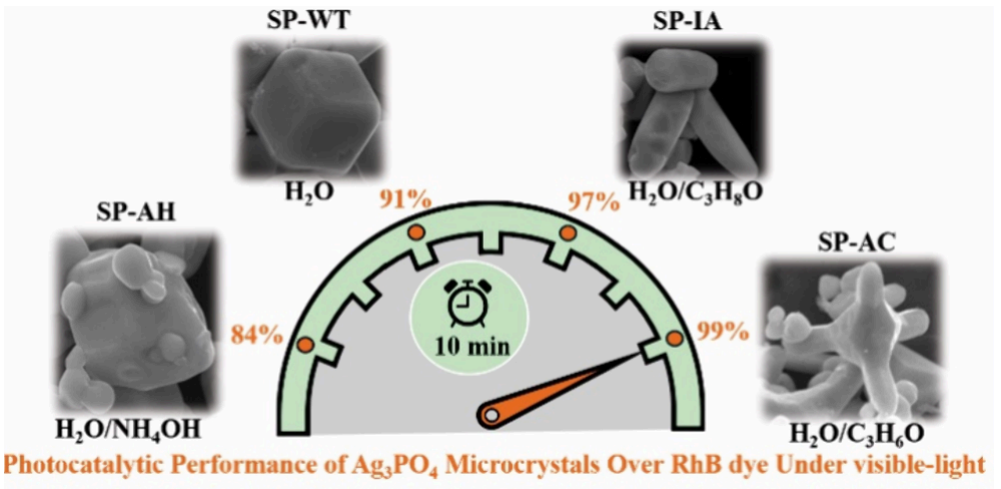

The design of particle size and morphology are a promising approach to investigating the properties exhibited by different types of materials. In the present study, the silver phosphate microcrystals (Ag_3_PO_4_) were first time synthesized using the hydrothermal and solvothermal method by combination of the solvents water/isopropyl alcohol (SP-IA), water/acetone (SP-AC), water/ammonium hydroxide (AP-AH), all in a ratio of 1:1 (v/v). The synthesized materials were structurally characterized by X-ray diffraction (XRD), Rietveld refinement, and Raman vibrational spectroscopy, where it was confirmed that the pure phase was achieved for all prepared samples. The study of the optical properties by UV–vis diffuse reflectance spectroscopy (UV–vis/DRS) and colorimetry revealed that the obtained materials have an optical bandgap between 2.30 and 2.32 eV. The FE-SEM images collected revealed different morphologies for the synthesized materials, with a predominance of tetraploid-shaped microcrystals for the SP-AC sample, rods for the SP-IA sample, cubes and polyhedral for the SP-WT sample and condensed polyhedral for the SP-AH sample. The photocatalytic performance against the Rhodamine B dye (RhB) was 100%, 98.2%, 94.2%, and 87.8%, using the samples SP-AC, SP-IA, SP-WT, and SP-AH as photocatalyst at time of 12 min. On the other hand, the antimicrobial performance of SP-AC sample showed superior performance, resulting in the minimum inhibitory concentration—MIC of 7.81 μg mL^–1^ for the strain of *E. coli*, 7.81 μg mL^–1^ for the strain of *E. aureus*, 15.62 μg mL^–1^ for the strain of *P. auruginosa*, and 15.62 μg mL^–1^ for the strains of *C. albicans*. In this way, was synthesized a promissory antimicrobial and photocatalyst material, through an easy and cost-effective method.

## 1 Introduction

In the past decades, the discussions about the preservation of ecosystems, as also, the effect of climate changes, has led to the important discussion about the future of world population.^1−4^ In this context, the search for efficient methodologies for monitoring and remediating emerging pollutants, mainly pharmaceuticals, pesticides, hormones, plastic derivatives and textile dyes, has frequently been reinforced by government actions.^5−7^ These residues exhibits a high contaminating potential that these compounds represent for the quality of water, soil and air, among other, reduction of photosynthetic processes in aqueous media, food contamination, microbiological resistance and the bioaccumulation of their metabolites.^8^

Once released into the natural environment, mainly due to the intense activity of the agroindustry and pharmaceutical industry, they induce the genetic modification of different types of microorganisms, such as fungi, bacteria and viruses.^9,10^ Therefore, both persistent organic effluents and multiresistant microorganisms are objects of current environmental problems, which seriously compromise the future of the next generations and the maintenance of species in the most different ecosystems.^11,12^

The World Health Organization (WHO) confirms that contamination by resistant microorganisms was responsible for 1.27 million deaths worldwide in 2019, and that the projection made by the world bank for the additional costs of maintaining global public health, will reach the US$ 1 trillion mark by the year 2050.^13^ Among the multiresistant pathogenic microorganisms, there are urgent threats the microorganisms *Carbapenem-resistant Acinetobacter*, *Candida auris* (*C. auris*), *Clostridioides difficile* (*C. difficile*), *Carbapenem-resistant Enterobacteriaceae* (CRE), and *Drug-resistant Neisseria gonorrheae* (N. gonorrheae). While the serious threats included the *Drug-resistant Candida*, *Multidrug-resistantPseudomonas aeruginosa* (*P. aeruginosa*), *Methicillin-resistantStaphylococcus aureus* (MRSA), *Drug-resistantStreptococcus pneumoniae* (*S. pneumoniae*), and *Drug-resistant Tuberculosis* (ALSO).

Aiming the remediating of effluents containing persistent organic pollutants, as also, the microbiological disinfection, technological approaches have been investigated, including the use of species with high oxidizing potential and metallic nanoparticles, have become promising for these purposes.^12,14,15^ In this way, Chairungsri et al.^16^ immobilized titanium dioxide nanoparticles composed of rutile and anatase on spherical glass substrates, followed by application in the decolorization of simulated aqueous solutions with direct blue, direct red and direct brown textile dyes, irradiated with UVA radiation lamps, reaching a percentage of degradation of pollutants close to 65% after 240 min of exposure. On the other hand, Gholami et al.,^17^ reported the synthesis, characterization and application of copper-doped zinc oxide nanorods in the photodegradation of the herbicide Bentazon in aqueous medium (pH = 7), obtaining total degradation of the molecules in 50 min of exposure to ultraviolet radiation.

Therefore, the use of advanced oxidative processes (AOPs), which use species with oxidative potential, including hydroxyl radicals (HO^•^), superoxides (O_2_^•–^), and holes (*h*^+^), photogenerated in semiconductor materials in aqueous medium has been investigated around the past decades.^18,19^ Once in an aqueous medium, these radicals attack the organic chains of organic molecules or bacterial walls under low selectivity, resulting in the mineralization of high-weight molecules or inactivation of the basic functions of microorganism cells.^20−23^ The use of these compounds in reducing cancer cells has also been reported in the literature,^22^ becoming an innovative approach in the nonconventional treatment of tumor cells without radiopharmaceuticals or chemotherapy treatments.

The process of generating radials using inorganic semiconductors, mainly metal oxides, consists of absorbing electromagnetic radiation with photons of energetic magnitude equal to or greater than the energy between the valence (VB) and conduction (CB) bands of the semiconductor.^24^ The absorption of these photons promotes the excitation of electrons (*e*^–^) from BV to BC, resulting in the appearance of holes (*h*^*+*^) in BV.^25^ Therefore, the described process allows the reduction of O_2_ dissolved in the aqueous medium to superoxide radicals (O_2_^•–^), at the same time, the holes promote the oxidation of water molecules to protons (H^+^), hydroxyl ions (HO^–^), and hydroxyl radials (HO^•^).^26^ However, the physicochemical characteristics of the different semiconductors lead to specific applications due to properties of these materials’, mainly the optical, textural, and semiconductor properties.^17,25,27−29^

In the study reported by Mertah et al.,^30^ the antimicrobial and photocatalytic properties of Cu/TiO_2_/Ag_3_PO_4_ heterojunction have been investigated, obtaining promising results in the selective reduction of nitrates as well as inhibition of bacterial strains *Staphylococcus aureus* (*S. aureus*) and *Escherichia coli* (*E. coli*). Michele et al.^23^ also reported the antimicrobial properties of heterojunction Ag/Ag_3_PO_4_ in their study, decorated in hydroxyapatite and calcium carbonate matrices, resulting in the synergistic effect for the inhibition of bacterial growth of the strains *S. aureus* and *Pseudomonas aeruginosa* (*P. aeruginosa*).

Although the photocatalytic properties of titanium oxide and zinc oxide have already been described in the literature, the limitations of these materials to ultraviolet region have led to the search for materials that allow the use of visible radiation from solar sources. Therefore, silver-based compounds, such as silver phosphate (Ag_3_PO_4_),^31−33^ silver vanadate (Ag_3_VO_4_),^34,35^ silver tungstates (Ag_2_WO_4_),^36,37^ silver molybdates (Ag_2_MoO_4_),^18,38^ silver niobates (AgNbO_3_),^39,40^ arise in this context, which exhibit strong absorption of visible light, and consequently the generation of radicals.

Among these reported semiconductors, the silver phosphate (Ag_3_PO_4_) is promissor n-type semiconductor^41^ which has been extensively studied in recent years due to its excellent optical, semiconductive, and photochemical properties, which allow effective applications in microbiological inhibition, degradation of POPs in aqueous medium and water splitting.^41^ Structurally, the Ag_3_PO_4_ has a cubic structure with space group of *P43̅n*, which are clustered [PO_4_] of tetrahedral symmetry, with P–O bond length of approximately 1,539 Å, and clusters [AgO_4_] of tetrahedral symmetry, however, distorted, exhibiting Ag–O bonds with lengths of 2.379(3) Å. The structural arrangement of silver phosphate, as well as its optical properties, allow the strong absorption of visible light, mainly due to the small bandgap value, close to 2.4 eV, associated with the electronic transitions between Ag (4d) and O(2p) orbitals, present in the valence band (VB) to Ag (5s and 5p) and P (3s) orbitals arranged in the conduction band (CB) thus, giving it the intense yellow color, characteristic of this material.^42^

Several methodologies have been used to obtain silver phosphate with size and morphologies by the literature,^28^ resulting in materials with distinct optical, structural, morphological, and semiconductor properties. Among the synthesis methods commonly employed, it is possible to highlight the chemical methods in an aqueous medium based on different precursors of silver ions and phosphorus ions. Thus, the use of chemical route such as hydrothermal,^25^ solvothermal,^43^ microwave,^32^ sol–gel,^30^ and sonochemistry^24^ methods are classically described in the literature.

In this context, silver phosphate nanocrystals in the form of polyhedral-like morphology were efficiently synthesized by Santos et al.,^44^ using the coprecipitation method in aqueous medium under vigorous magnetxic stirring, starting from the precursors silver nitrate (AgNO_3_) and dibasic sodium phosphate (NaH_2_PO_4_). In their study, Dong et al.^45^ presented the control of morphology and size of crystals using the chemical precipitation method with the addition of polyvinylpyrrolidone surfactant (PVP), obtaining particles with rhombohedral morphology, as well as nanorods, with dimensions between 1 and 2 μm in length and 250–450 nm in diameter. In the study conducted by Hsieh et al.,^26^ microcrystals with cube-shaped morphologies, Rhombic dodecahedra, {100}-truncated rhombic dodecahedra, tetrahedra, and tetra-pods were synthesized without the addition of surfactants by the chemical coprecipitation method, with superior photocatalytic properties for microcrystals with cube-shaped morphology. On the other hand, Botelho et al.^46^ reported the variation of surface energy for different surfaces exposed to silver phosphate microcrystals, which are completely related to the photocatalytic and antimicrobial properties; according to the authors, the {100} face is the one that exhibits the highest surface energy, which theoretically enables the best performance in oxidative processes. Thus, the study of the design and morphology control of silver phosphate crystals has become one of the focuses of research that seeks to improve the physicochemical, optical, semiconductor, photocatalytic, and antimicrobial properties of silver phosphate nano and microcrystals.

Based on the above, the present study reports the effect of hydrothermal synthesis on silver phosphate microcrystals morphology, using the water, and mixture of solvents water/acetone, water/isopropyl alcohol, and water/ammonium hydroxide (1:1 V/V) under 120 °C for 24 h. The materials were characterized by structural (XRD, Raman), optical (UV–vis/DRS and Colorimetry), and morphological (SEM) analysis, as well as the photocatalytic performance in the degradation of the RhB dye in an aqueous medium under blue LED light. While the antimicrobial performance, was investigated against the bacterial strains *E. coli*, *P. auruginosa*, *S. aureus*, and fungus *C. albicans*.

## 2 Materials and Method

### 2.1 Materials

Silver nitrate, AgNO_3_ (Sigma-Aldrich, purity ≥ 99.0%); sodium phosphate monobasic, NaH_2_PO_4_ (Sigma-Aldrich, purity ≥ 99.0%); acetone, CH_3_COCH_3_ (Sigma-Aldrich, purity ≥ 99.5%); ammonium hydroxide, NH_4_OH (Sigma-Aldrich, purity = 28–30%); isopropyl alcohol, (CH_3_)_2_CHOH (Sigma-Aldrich, purity ≥ 98%); rhodamine b dye, C_28_H_31_ClN_2_O_3_ (Sigma-Aldrich, purity ≥ 99.5%); and distilled water were used in the synthesis or photocatalytic experiments.

### 2.2 Synthesis of Silver Phosphate

**Figure 1:**
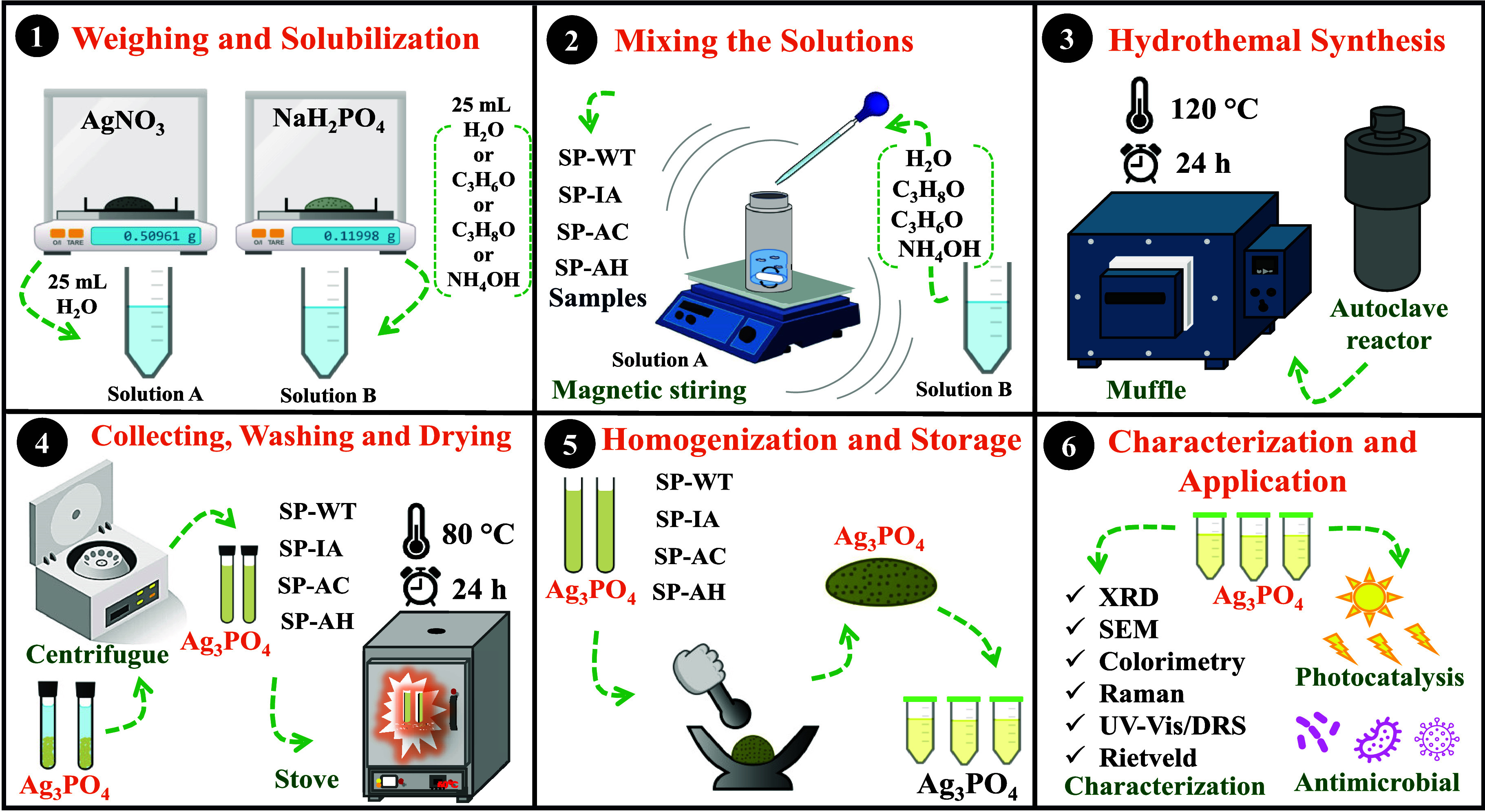
Step-by-step adopted in the synthesis of SP-WT, SP-IA, SP-AC, and SP-AH samples.

Silver phosphate was synthesized by conventional hydrothermal method modifying the steps reported by Wang et al.,^28^ as can be seen in Figure 1. Typically, 3 mmol of silver nitrate was first solubilized in a Falcon tube (50 mL capacity) with 25 mL of ultrapure water under vortex stirring, which was identified as solution A. On the other hand, 1 mmol of sodium phosphate monobasic was added in 25 mL of ultrapure water in another Falcon tube with 50 mL capacity and solubilized by vortex stirring, thus identified as solution B. Then, solution A was transferred to a stainless-steel autoclave system, coupled with a Teflon cup (100 mL capacity), and magnetically stirred. In contrast, solution B was gently drop-by-drop added to solution A, in which a yellow suspension was quickly formed. The suspension was magnetically stirred for 10 min after the total addition of solution B into solution A. The obtained suspension was sealed and heated at 120 °C for 24 h, then cooled to room temperature, collected by centrifugation (5000 rpm for 5 min), washed several times with distilled water, and dried at 80 °C for 24 h. The sample was labeled as SP-WT.

A similar procedure was adopted in the synthesis of samples with different solvents. However, a certain volume of each solvent was added to the solubilization of reactants. Therefore, 3 mmol of silver nitrate was solubilized in 25 mL of solution distilled water to obtain the solution A, while the solution B was prepared by addition of 1 mmol of sodium phosphate dihydrate in 25 mL of solution acetone/water 1/1 (v/v). Solution B was dropwise added into solution A under magnetic stirring, which remained under stirring for 10 min. The suspension was transferred to a Teflon cup, sealed into a stainless-steel autoclave and, heated at 120 °C for 24 h, cooled to room temperature, collected by centrifugation (5000 rpm for 5 min). After, it was washed several times with distilled water and dried at 80 °C for 24 h. The sample was labeled as SP-AC. A similar procedure was adopted for other samples, changing the solvent for isopropyl alcohol (at the same proportion solvent/water) or ammonium hydroxide, where the obtained samples were labeled SP-IA and SP-AH, respectively.

### 2.3 Characterization

#### 2.3.1 X-ray Diffraction

The X-ray diffraction pattern of each sample was collected by Shimadzu diffractometer, XRD 7000 with copper anode (CuKα = 0.15406 nm) adopting a step of 0.02°, scan velocity of 2° min^–1^ in 2θ from 10 to 100°.

#### 2.3.2 Vibrational Raman Spectroscopy

The Raman spectra were recorded at room temperature operating a Bruker Raman confocal microscope, SENTERRA II, equipped with a charge-coupled device (CCD) detector. For all samples, the vibrational spectrum was collected using a green laser with a wavelength of 532 nm, an output power of 0.25 mW, 30 coadditions, and an integration time of 10 s^–1^ from 85 to 1500 cm^–1^.

#### 2.3.3 Ultraviolet–Visible by Diffuse Reflectance Spectroscopy (UV–vis/DRS)

The DRS spectrum for all samples (powder) was recorded from 190 to 800 nm, using a Shimadzu spectrophotometer, UV2600i, coupled with a diffuse reflectance sphere, in which the DRS data was collected at a scan rate of 5 nms^–1^, where the barium sulfate (BaSO_4_, Sigma-Aldrich, purity > 99.9) was used as reflection standard.

#### 2.3.4 Colorimetric Analysis

The color of the samples was acquired by colorimetric analysis operating a Delta Vista spectrophotometer 450 G, collecting the reflectance against wavelength data from 400 to 700 nm with wavelength step of 10 nm, in which the parameters of CIELab a*, b*, c*, L* (luminosity), and H were obtained.

#### 2.3.5 Scanning Electron Microscopy (SEM)

The morphology of silver phosphate crystals was investigated by field emission scanning electron microscopy through an FEI Quanta FEG250 microscope operating at accelerating voltage and currents of 15 kV and 30 kV, respectively. Before the analysis, a small part of the samples (∼30 mg) was dispersed in acetone (2 mL) and sonicated for 3 min, then the suspension was drop-by-drop deposited on the aluminum paper over the stub surface.

#### 2.3.6 Photocatalytic Experiments

The photocatalytic performance of the synthesized samples was investigated in the decolorization of the RhB dye under radiation with a wavelength in the visible region (λ_max_ = 425 nm), using a system developed by the authors themselves, which consisted of a box made of acrylic, featuring 9 (nine) LEDs with a power of 3W each, arranged in an area of 10 cm^2^, two microfans for cooling the system and a pneumatic pump with a flow rate of 1.5 Lmin^–1^, for oxygenating the solution. The kinetics of degradation for RhB dye solution was monitored by a decrease of λ_max_ of RhB at 554 nm, characteristic of the chromophore group, from 1 to 20 min. Therefore, 0.5 mL was collected and centrifugated, and the supernatant was analyzed in the UV–vis 2600i spectrometer at a scan rate of 20 nms^–1^ from 190 to 900 nm. The degradation was estimated by eq 1 as given:
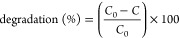
1where *C*_0_ is the initial concentration of RhB dye (5 mg L^–1^) and *C* is the concentration at different times (*t*).

#### 2.3.7 Antimicrobial Assay

The microdilution technique (CLSI, 2017–with adaptations) was used, reducing resazurin for antibacterial tests and reducing 2,3,5-triphenyltetrazoic chloride (TTC) for antifungal tests. The samples were tested against the strains commercially acquired from Cefar Diagnóstica Ltd.a: *Staphylococcus aureus* (CCCD-S009), *Escherichia coli* (CCCD-E005), *Pseudomonas aeruginosa* (CCCD-P004) and *Candida albicans* (CCCD-CC001). 96-well microplates were used, where 100 μL of the sample and 100 μL of the microbial inoculum were pipetted. The positive control for the bacteria was Levofloxacin at 0.25 mg/mL, and for the fungi, Terbinafine at 0.40 mg/mL. As a negative control, only the microbial inoculum was inserted. For sterility control, 100 μL of the sterile culture medium was used to prepare the inoculum (Mueller Hinton broth for the bacteria and Sabouraud broth for the yeast). Subsequently, the plates were incubated at 37 °C for 24 h (bacteria) and 48 h (fungi) in a BOD oven. After adding the developers, the plates were incubated again at 37 °C for 1 to 2 h to verify the color change as a result of the reduction of the developers. The Minimum Inhibitory Concentration (MIC) was determined by successive dilutions of the samples. The lowest concentration that inhibited microbial growth was considered MIC.

## 3 Results and Discussion

### 3.1 X-ray Diffraction and Structural Rietveld Refinement

**Figure 2:**
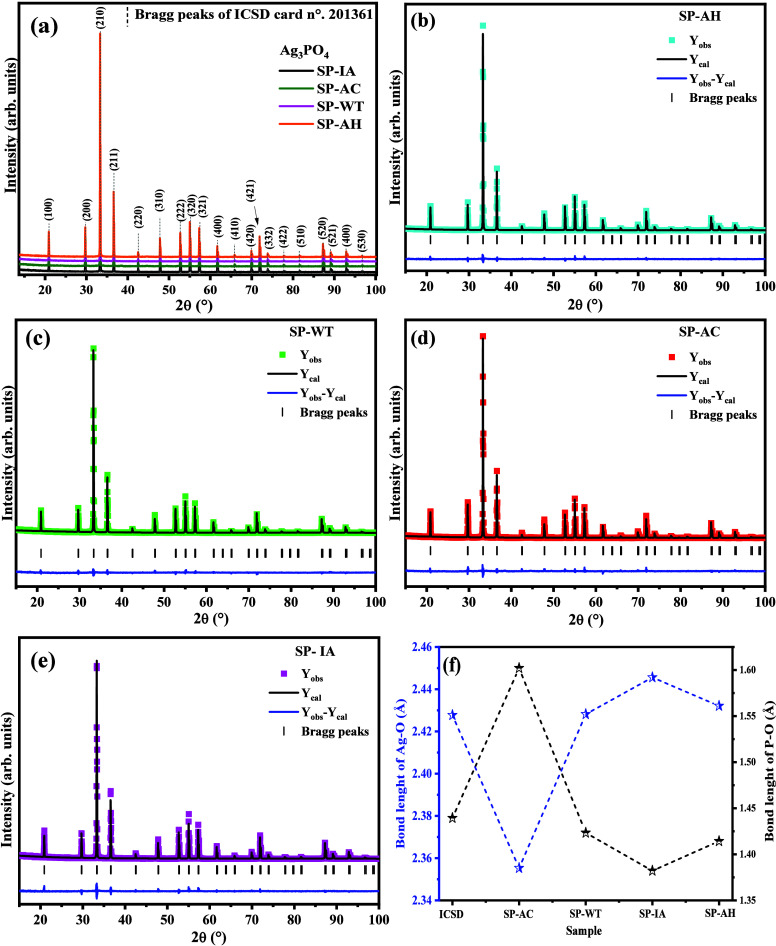
(a) X-ray diffraction pattern of all samples, structural Rietveld refinement of (b) SP-AH, (c) SP-WT, (d) SP-AC, and (e) SP-IA, and (f) the Ag–O and P–O bond length for all refined structures.

The diffraction pattern, structural Rietveld refinement plot, and the variation of the Ag–O and P–O bond length are shown in parts of Figure 2(a–f) of all synthesized samples. The indexing of the crystallographic planes in the diffraction patterns of the obtained samples (Figure 2a) revealed the formation of the cubic structure phase of the space group *P4̅3n* and two formulas per unit cell (*Z* = 2), all of which agree with those contained in the Inorganic Crystal Structure Database (ICSD) card no. 201361^47^ and 14000.^25,46,48,49^ In addition, it is possible to note that the intensity and profile of the diffraction peaks suggest the obtaining of materials with a high degree of crystallinity and short- and long-range ordering, with no appearance of peaks associated with the formation of secondary phases, traces of precursors and/or formation of metallic silver.^44^ Therefore, the efficiency in obtaining materials by the proposed synthesis method was confirmed, resulting in high-purity samples.

Variations in the intensity and graphic profile of the crystallographic planes were observed when normalizing and superimposing the diffraction patterns of all samples. These variations were studied using structural refinement by the Rietveld method,^50^ which allowed a detailed study of the network parameters (*a*, *b*, *c*, α, β, γ), atomic coordinates (*x*, *y*, *z*), Unit Cell Volume (*V*), Occupation of atoms (*O*_cc_), and the orientation of crystallographic planes.^51−53^

The Rietveld refinement was performed by computing the data using the Fullprof software September 2023 for Windows, adopting the Thompson–Cox–Hastings pseudo-Voigt (Axial divergence asymmetry) function.^54^ On the other hand, the background was refined using a sixth-order polynomial, where the quality of the refinement was accompanied by the values of quality parameters *R* (*R*_exp_, *R*_p_, *R*_wp_, and χ^2^), as well as the visual difference contained in the residual line (residual = *Y*_obs_ – *Y*_cal_). For all cases, the value of χ^2^ ≤ 2 indicates a good quality refinement.

Figure 1(b–d) shows the graphs for the structural refinement performed for the samples SP-AH, SP-WT, SP-AC, and SP-IA, where *Y*_obs_ are the experimental values and *Y*_cal_ are the values calculated from the adjustment obtained for each refined parameter. The vertical lines between the obtained plots and the residual line indicate the Bragg positions for the crystallographic plane’s characteristic of the cubic structure of silver phosphate. Therefore, there was an excellent agreement between the theoretical and experimental data, and there was no occurrence of crystallographic planes associated with secondary phases, confirming the samples’ purity. In addition, the detailed study of the lattice parameters revealed that the volume of the unit cell obeyed the descending order of 217.43(6)Å^3^ (SP-WT) > 217.40(4) Å^3^ (SP-AC) > 217.40(8) Å^3^ (SP-AI) > 217.40(6) Å^3^ (SP-AH).

Figure 2(f) shows the length of the bonds between the Ag–O and P–O atoms present in the clusters [AgO_4_] and [PO_4_] of tetrahedral symmetry, where the most extended Ag–O binding length was verified for the SP-IA sample. In contrast, the most extended P–O binding length was identified for the SP-AC sample, and the lowest binding length was also associated with the same samples. The variation in the bond length in the clusters [AgO_4_] and [PO_4_] indicates changes in the crystallographic ordering and consequent morphological modifications of the crystals formed. The complementary information obtained by the structural refinement is summarized in Table S1, available in the Supporting Information, where it is possible to check the variations for the atomic position, sites, occupation (*O*_cc_), and *R* parameters of the quality of the refinement employed. It is important to note that for all refined samples, the value of χ^2^ was less than 2, indicating that the computed data are reliable and reproducible, with high similarity between theoretical and experimental information.

The size of crystallite (*D̅*_*hkl*_) of the synthesized samples was obtained by adopting the Scherrer model^55^ (eq 2), as follows:
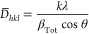
2Where *k* is the constant associated with the shape of the particles, in this case, an approximately spherical shape was adopted, i.e., *k* = 0.91, while λ, is the wavelength of the copper anode used in the X-ray diffractometer (λ = 0.15406 nm).^56^ On the other hand, θ corresponds to the diffraction angle related to the plane of greatest intensity (210), Indexed in 2θ near 31.2°, and β_Tot_, the width at half height of diffraction peaks (fwhm) corrected by lanthanum hexaboride diffraction pattern (LAB_6_, Sigma-Aldrich, purity >99.9%), using eq 3.
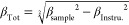
3Where sample is the contribution of the half-height width of the diffraction peaks of the sample (β_sample_), while β_instru_. is an instrumental contribution on the width to full width at half-maximum of diffraction peaks. From the results, it can be seeing a decrease of crystallite size for the SP-AC, SP-IA, SP-AH, and SP-WT samples, respectively 121, 118, 119, and 112 nm. Based on these results, it is noted that the presence of organic solvents in the reaction medium directed the growth of microcrystals, probably due to the increase in the pressure of the reaction system and the surfactant effect of the carbon chains. This behavior was also reported by Cunha et al.^57^ using alcohols with different lengths of carbon chains, i.e., an increase in the nonpolar character, which increased the size of microcrystals when the morphology of the materials was investigated by scanning electron microscopy (SEM).

**Figure 3:**
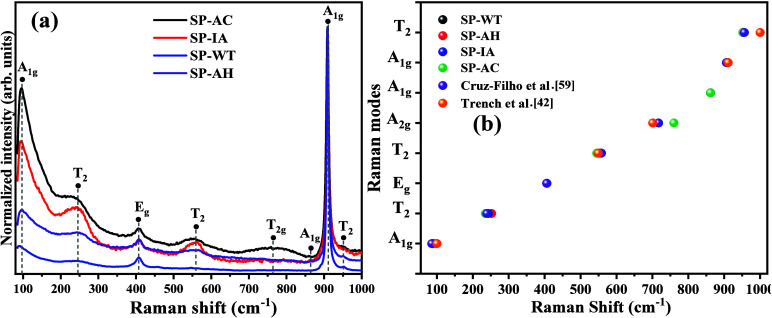
Raman spectrum (a) samples SP-AH, SP-WT, SP-AC, and SP-IA, and (b) the band position for all active modes in the region from 80 to 1000 cm^–1^, compared with the Cruz-Filho et al.^59^ and Trench et al.^42^

The vibrational information on the synthesized samples was investigated by Raman vibrational spectroscopy, as seen in parts of Figure 3(a, b). The literature reports 48 degrees of freedom for the cubic structure of space group silver phosphate *P4̅3n* and punctual group *T*_*d*_^4^, related to the 16 atoms present in the primitive unit cell, of which the sites for the atoms of silver (Ag), phosphorus (P), and oxygen (O), are S4, T, and C3, respectively in the center of the Brillouin zone (point).^49^ Although the group theory reveals 18 optical modes and three acoustic modes, as presented in eqs 4 and 5.
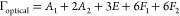
4
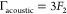
5However, among the optical modes for the structure of silver orthophosphate, only 18 are active modes, six of which are active in infrared spectroscopy (Γ_IR_ = 6*F*2), and ten active modes in Raman spectroscopy (Γ_Raman_ = *A*_1_ (ν_1_) + 3*E*(*T*, 2ν_2_) + 6*F*_2_ (3*T*, *L*, ν_3_, ν_4_).^49,58^ Of the active modes in Raman spectroscopy, eight of the ten vibrational modes are generally reported, with the remaining two being difficult to identify due to the low relative intensity in the experimental spectrum for silver phosphate.

As seen in Figure 3(a,b), the analysis of the spectrum obtained for the SP-WT sample provided the identification of five active modes in the 90 wave numbers (A_1g_), 238 (T_2g_), 407 (E_g_), 909 (A_1g_), and 950 cm^–1^ (T_2_). In contrast, for the SP-IA and SP-AC samples, seven bands out of 95 were identified (A_1g_), 243 (T_2g_), 405 (E_g_), 558 (T_2_), 861 (A_1g_), 904 (A_1g_), and 950 (T_2_) cm^–1^. For the SP-AC sample, the eight active modes were identified, which were wave numbers 94 (A_1g_), 242 (T_2g_), 406 (E_g_), 555 (T_2_), 764 (T_2g_), 863 (A_1g_), 909 (A_1g_), and 959 (T_2_) cm^–1^. For all cases, vibrational modes situated close to 90 and 238 cm^–1^ are derived from the external movements of the structure, as well as rotational, from the P–O bonds present in the clusters [PO_4_] of tetrahedral symmetry. In the wave numbers close to 407 and 558 cm^–1^, they are associated with the bending movements of the groups [PO_4_], while the modes located in 764 and 863 cm^–1^, are due to the symmetrical stretches of the P–O–P bonds in the clusters [PO_4_]. Finally, the active modes 909 and 959 cm^–1^ are characteristic of asymmetric stretches of the P–O–P connections of the clusters [PO_4_] of tetrahedral symmetry.

All the vibrational modes identified in the spectra collected for the synthesized samples are in excellent agreement with those reported by the literature.^27,59^ However, there are small differences in the position of the bands associated with the active vibrational modes due to the synthesis method, experimental conditions, and types of reactants used in the syntheses. In addition, in Figure 3(b), it is possible to visualize the positions of the bands identified for the active vibrational modes of the synthesized structures with those reported by Trench et al.^42^ and Cruz-Filho et al.^59^ Therefore, it is possible to confirm that the vibrational modes identified agree with the literature consulted, corroborating the analysis presented in the structural analysis by XRD. The variations observed in the samples regarding the intensity of the crystallographic planes extend to the differences observed for the presence of the bands associated with the active modes of each sample, as reported in Raman vibrational spectroscopy.

The optical properties of the synthesized samples were studied using ultraviolet–visible spectroscopy (UV–vis) by diffuse reflectance (DRS). In this sense, diffuse reflectance spectra (*R*%) were initially collected as a function of wavelength (λ), as seen in Figure S1, available in the Supporting Information. Based on the reflectance spectra presented, it is possible to highlight the region between 200 and 400 nm, i.e., the ultraviolet region, which exhibits a wide band, indicating that it is the region with the greatest absorption of incident light. It is suggested that the variation in the absorbance values in this region may be related to the quantum confinement of the particles in the formation of the crystals of the structure, where the effect of pressure and temperature promoted by the different solvents adopted directly implied the structural, vibrational, and optical properties.^60^

The optical bandgap (*E*_gap_) was obtained by the Kubelka–Munk method,^61^ mathematically represented by eqs 6 and 7, where *F*(*R*_∞_) corresponds to the Kubelka function. At the same time, *K* and *S*, are the scattering absorption coefficients, respectively.^62^ The term *R*_∞_, is the infinitesimal reflectance for a thin layer of material, where *R*_∞_ = *R*_sample_/*R*_standard_, where *R*_standard_ was obtained for barium sulfate, BaSO_4_ (Sigma-Aldrich, purity >99.9%), type as a reflectance standard in DRS spectroscopy.
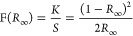
6
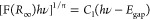
7Where *h* corresponds to Planck’s constant (*h* = 4.136 × 10^–15^ eV.s), and *n* corresponds to the type of electronic transitions, respectively. In this case, the electronic transitions adopted by the literature consulted^29,43,63^ were of the type allowed indirect, *n* = 2.^25^

Based on the theoretical study by density of occupied states (DOS), carried out independently by the authors Botelho et al.^46^ and Liu et al.,^31^ the main contributions of the valence band (BV) in the electron transitions come from the O 2p and Ag 4d states, while the P 3p and O 2s orbitals, although present in the VB, exhibit a small contribution in the electron transitions compared to the others. Above the VB and lower than the Fermi level, the contributions continue to be mostly from the O 2p and Ag 4d orbitals; however, on a greater dispersion of the O 2p orbitals, which makes the electronic transitions between the valence band and the conduction band (CB) more effective. Above the Fermi level, it is observed that the contributions are purely associated with the Ag 5s and Ag 5p orbitals, which give rise to the antiligand states.

**Figure 4:**
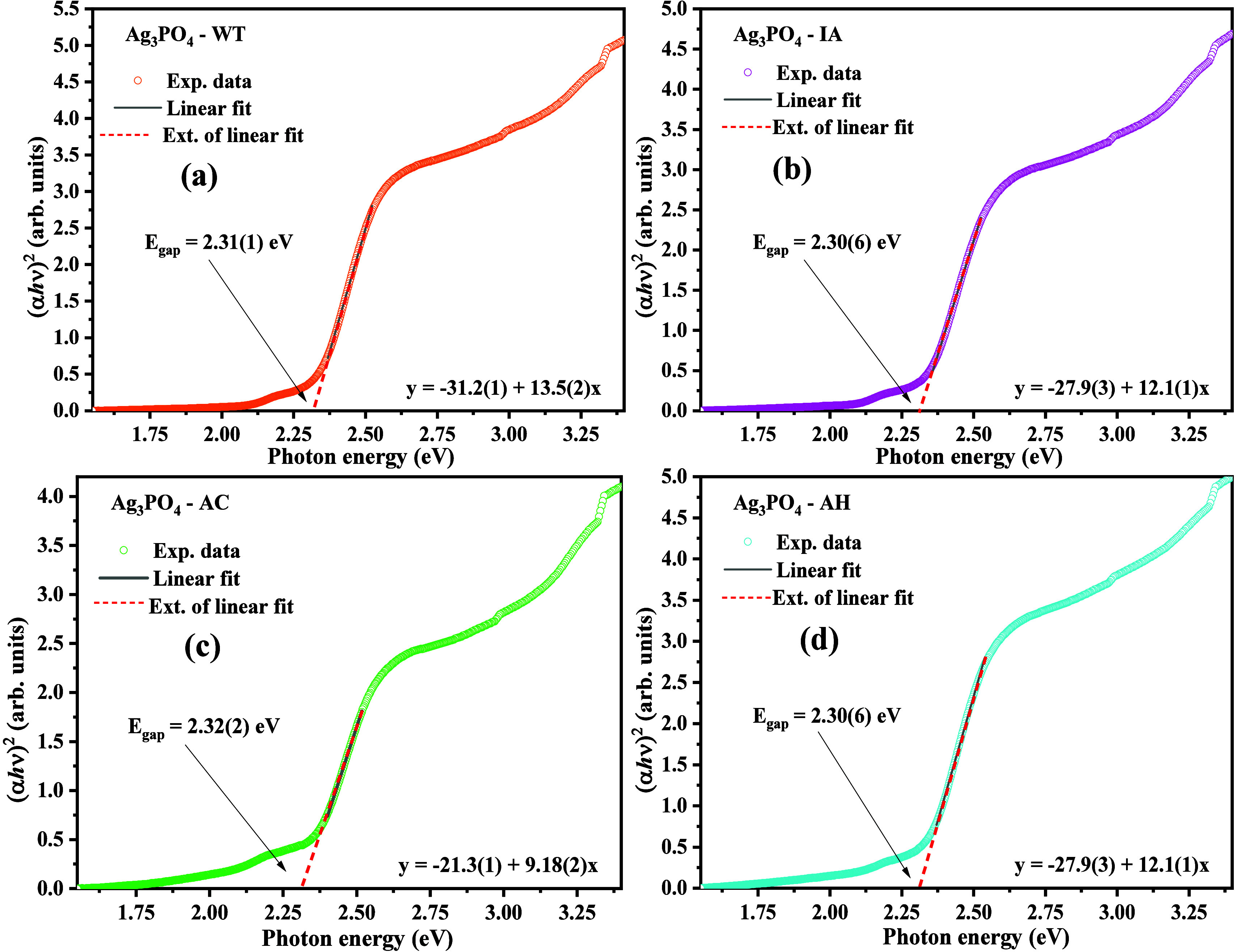
Tauc plot for optical bandgap of (a) SP-WT, (b) SP-IA, (c) SP-AC, and (d) SP-AH.

In the determination of the optical bandgap of the materials, the extrapolation of the straight section of the curve obtained for the plot of the [*F*(*R*_∞_)*h*ν]^1/2^ (*y*-axis), versus photon energy is usually adopted, which was obtained by converting the wavelength values using Planck’s equation (*E*_phot_ = 1240/λ). Figure 4(a–d) shows the plots for the modified Kubelka–Munk function, [*F*(*R*_∞_)*h*ν]^1/2^, as a function of the photon energy (*h*), for the νsamples SP-WT, SP-IA, SP-AC, and SP-AH, as well as the equations obtained for the extrapolation of the straight section of the curve to obtain the value of *E*_gap_.

The values of E_gap_ for the SP-WT, SP-IA, SP-AC and SP-AH samples, as shown in Figure 4(a–d), they were 2.31(1), 2.30(6), 2.32(2), and 2.30(6) eV, respectively. Therefore, it is noted that the SP-IA and SP-AH samples resulted in similar values, with the latter being the lowest value of *E*_gap_, while the lowest value was obtained for the SP-AC sample. The results confirmed the strong absorption in the visible spectrum of light region, which allows electronic transitions between the BV and BC bands, by absorbing radiation with a wavelength close to 540 nm. All these values agree with those reported in the literature,^6,31,46^ using different synthesis methods. These differences are associated with structural defects, oxygen vacancies, and particle size, agreeing with the information presented in the XRD and Raman spectroscopy characterization.

From the *E*_gap_ values obtained DRS spectroscopy, the energy values for the valence bands (*E*_VB_) and of conduction (*E*_CB_) were calculated, adopting eqs 8 and 9. Where χ is the total electronegativity for Ag_3_PO_4_(χ_Ag_3_PO_4__) = 5.959), *E*° corresponds to the energy of the free electron (*E*° = 4.5 eV) and *E*_gap_ the energy of the bandgap for each synthesized sample.
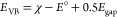
8
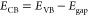
9Due to the small difference between the bandgap values for the SP-WT, SP-IA, SP-AC, and SP-AH samples, the band position values for *E*_VB_ were 2.61, 2.60, 2.62, and 2.60 eV, respectively. The respective *E*_CB_ values were 0.304, 0.310, 0.300, and 0.31 eV. These results suggest that the optical properties of silver phosphates provide the opportunity for the oxidation of water molecules, since the value of *E*_VB_ is greater than the potential for the pair *E*(O_2_/H_2_O), i.e., greater than 1.23 eV, through the holes by the photoexcitation of electrons to CB. That is, easy formation of holes (*h*^*+*^) after the absorption of photons with energy equal to or greater than the energy of the bandgap.^29^

The colorimetric properties of the samples SP-WT, SP-IA, SP-AC, and SP-AH were studied according to the colorimetric coordinate values of the CIELab system.^64^ Where color variations for the +a* coordinate reflect contributions associated with red color tones.^65^ In contrast −a* values indicate contributions related to green color. On the other hand, the colorimetric coordinates +b* and −b* are associated with the colors yellow and blue, respectively.^66^ The variations for the L* parameter indicate the illuminance, and have a range between 0 and 100, indicating dark tones for values close to 0, and light tones when close to 100.^67^ In addition, the parameters C* and H* are called chroma and Hue angle, respectively, and make up the components that indicate the position of the colorimetric and tristimulus coordinates for color and energy variation.^67^ This combination can be directly related to the RGB (Red–Green–Blue) coordinates and the hexadecimal system of color-specific components (HEX Code Colors).

Table 1 shows the values of colorimetric coordinates (L*, a*, b*, C*, and H*), as well as the HEX codes and coordinates of the RGB system for the samples SP-WT, SP-IA, SP-AC, and SP-AH. In addition, the reflectance spectra obtained in the colorimetric study are available in Figure S2, available in the Supporting Information.

**Table 1:**
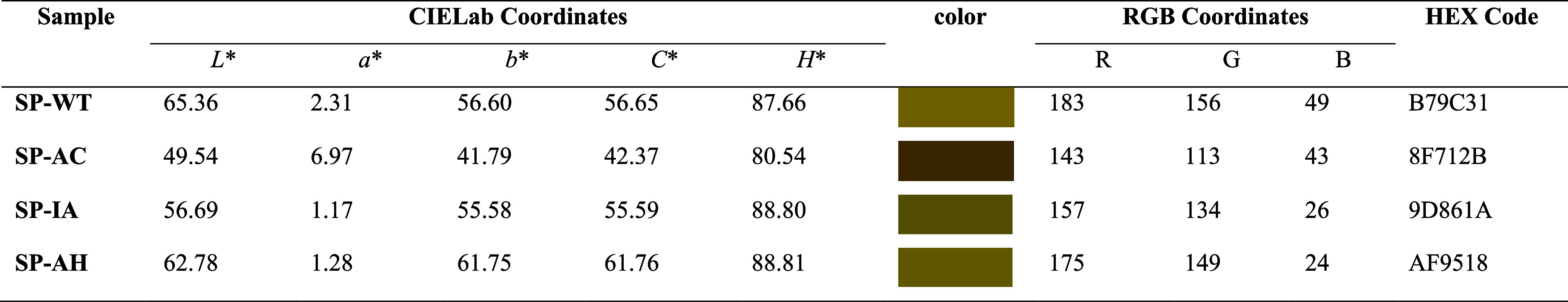
Colorimetric Coordinates, RGB, and HEX Code for SP-WT, SP-IA, SP-AC, and SP-AH Samples

Based on the results obtained, it is noted that the decreasing order of clarity, i.e., luminance, for the samples obtained was: SP-WT (L* = 65.36) > SP-AH (L* = 62.78) > SP-IA (L* = 56.69) > SP-AC (L* = 49.54), which may be related to the morphology of the structures formed, as well as the occurrence of formation and anchorage of metallic nanoparticles on the surface of the structures, and oxygen vacancies.^44,48^ The trend observed for the percentage reflectance in the graph available in Figure S2 agrees with the UV–vis/DRS spectra, presented in Figure S1, and the SP-WT sample was the one that exhibited the lowest absorption intensity, consequently, the highest percentage of reflectance, in the region between 400 and 800 nm. All synthesized samples exhibited a yellow color pattern, especially with distinctly different tones, i.e., different luminance values.

**Figure 5:**
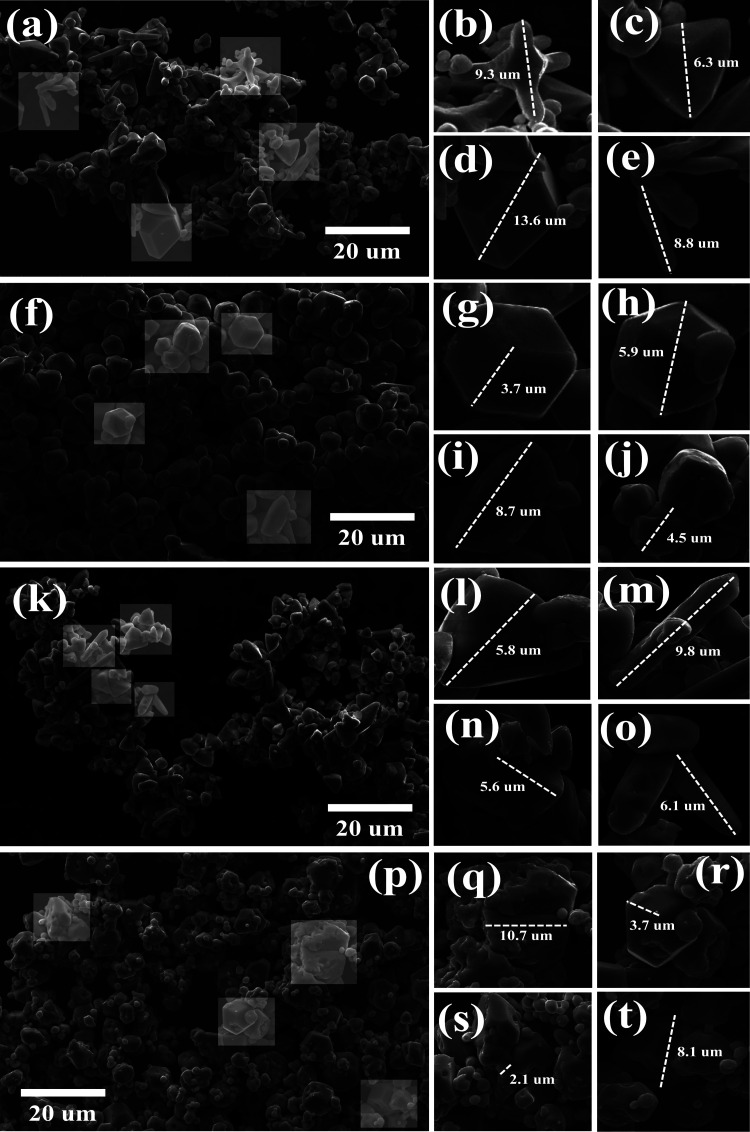
SEM images of (a–e) SP-AC, (f–j) SP-WT, (k–o) SP-IA, and (p–t) SP-AH.

The morphological study of the structures that make up the samples SP-WT, SP-IA, SP-AC, and SP-AH was done by scanning electron microscopy (SEM), as seen in parts of Figure 5(a–t).

The images collected for the SP-AC sample, presented in Figure 5(a–e), reveal microcrystals with different morphologies, which is easily identified as microcrystals shaped like tetrapods (Figure 5b), tetrahedral (Figure 5c), rhombic dodecahedrals (Figure 5d), and rods (Figure 5e), in this case, with an elongated shape. From the images shown in parts of Figure 5(a–e), it is possible to identify tetrahedral structures with rod-shaped ends, suggesting that the formation of tetrapod structures derives mainly from this morphology.

In the study conducted by Wang et al.,^28^ silver phosphate microcrystals were efficiently synthesized by the hydrothermal method, investigating the changes occurred by the addition of urea in the reaction medium, where the formation of elongated rod-shaped structures was obtained under 4 h time. In contrast, tetrapod-shaped structures were obtained at synthesis time above 6 h of hydrothermal processing. The authors also describe that the presence of urea in the reaction medium leads to an increase in the pH of the chemical medium with an increase in the reaction time, which induces an increase in the exposure of the {110} plane, conducing to the tetrapod structures growth. Morphological variations for silver phosphate microcrystals have also been reported by Hsieh et al.,^26^ adopting the hydrothermal method with the addition of different amounts of ammonium nitrate (NH_4_NO_3_), which resulted in the formation of microstructures with cubic shape, rhombic dodecahedral, rhombic dodecahedral with face exposure {100}, tetrahedrons and tetrapods. The authors identified that adding excess phosphate source to the reaction medium, in this case, potassium hydrogen phosphate (K_2_HPO_4_), led to the formation of tetrapod and tetragonal structures.

The microcrystals shown in the images in Figure 5(f–j), corresponding to the SP-WT sample, have a distinct morphology compared to the microcrystals obtained for the SP-AC sample. Where it is noticeable to distinguish rhombic dodecahedral structures, cubic structures and polyhedral with irregular faces with size distribution between 2 and 18 μm. These structures are commonly reported for silver phosphate when the hydrothermal method is adopted, using silver nitrate and mono- or dibasic sodium or potassium phosphate.^6,29,31,32,68^

The synthesis conducted by the mixture between the solvents distilled water and isopropyl alcohol (SP-IA), as shown in parts of Figure 5(k–o), reveals that the predominant morphology for the microcrystals formed is the tetragonal and elongated rods. Hsieh et al.^26^ presented the synthesis, characterization and photocatalytic application of silver phosphate microcrystals with cubic morphology, dodecahedrons, and tetrahedrals and reveal in their literature review that the addition of ethyl alcohol to the reaction medium leads to the formation of tetragonal structures. In this case, they are corroborating the results observed in this study for the addition of isopropyl alcohol to the reaction medium in the synthesis of silver phosphate microcrystals, in a 50/50 ratio (v/v).

The images presented in Figure 5(p–t), collected for the SP-AH sample, reveal the majority of polyhedra with a dodecahedron shape, which exhibits a high degree of superficial crystalline defects and microcrystals with cubic morphology. Based on the results obtained, as well as those reported in the literature,^28,46^ we suggest that the formation of the microcrystals that make up the SP-AH sample occurs by the initial complexation of silver ions (Ag^+^) for hydroxide ions (OH^–^), as well as ion interaction NH_4_^+^ with the ions H_2_PO_4_^–^. This process leads to a self-assembly of the particles to form the microcrystals, guided by the groups NH_4_^+^ and ^–^OH, available on the surface of the microcrystals. However, under the strong effect of the redissolution of the initially formed particles, by the effect of temperature and pressure characteristic of the solvothermal and hydrothermal methods.^6,32,43,48,59^ In this process, the accumulation and condensation of smaller particles is characteristic, resulting in particles with larger dimensions commonly reported to have a high density of defects for the microcrystals formed.

The photocatalytic performance of the synthesized samples was investigated in the discoloration of the RhB dye, analyzing the reduction of the characteristic band of the chromophore, at wavelength of 554 nm.^25,27,29,32,41,59^ The evolution of the solution decolorization process was monitored at intervals of 2 consecutive min, up to 20 min of exposure. For comparison, the test was performed without the catalyst (photolysis) and using titanium dioxide (TiO_2_, Degussa P25) as the standard photocatalyst.

**Figure 6:**
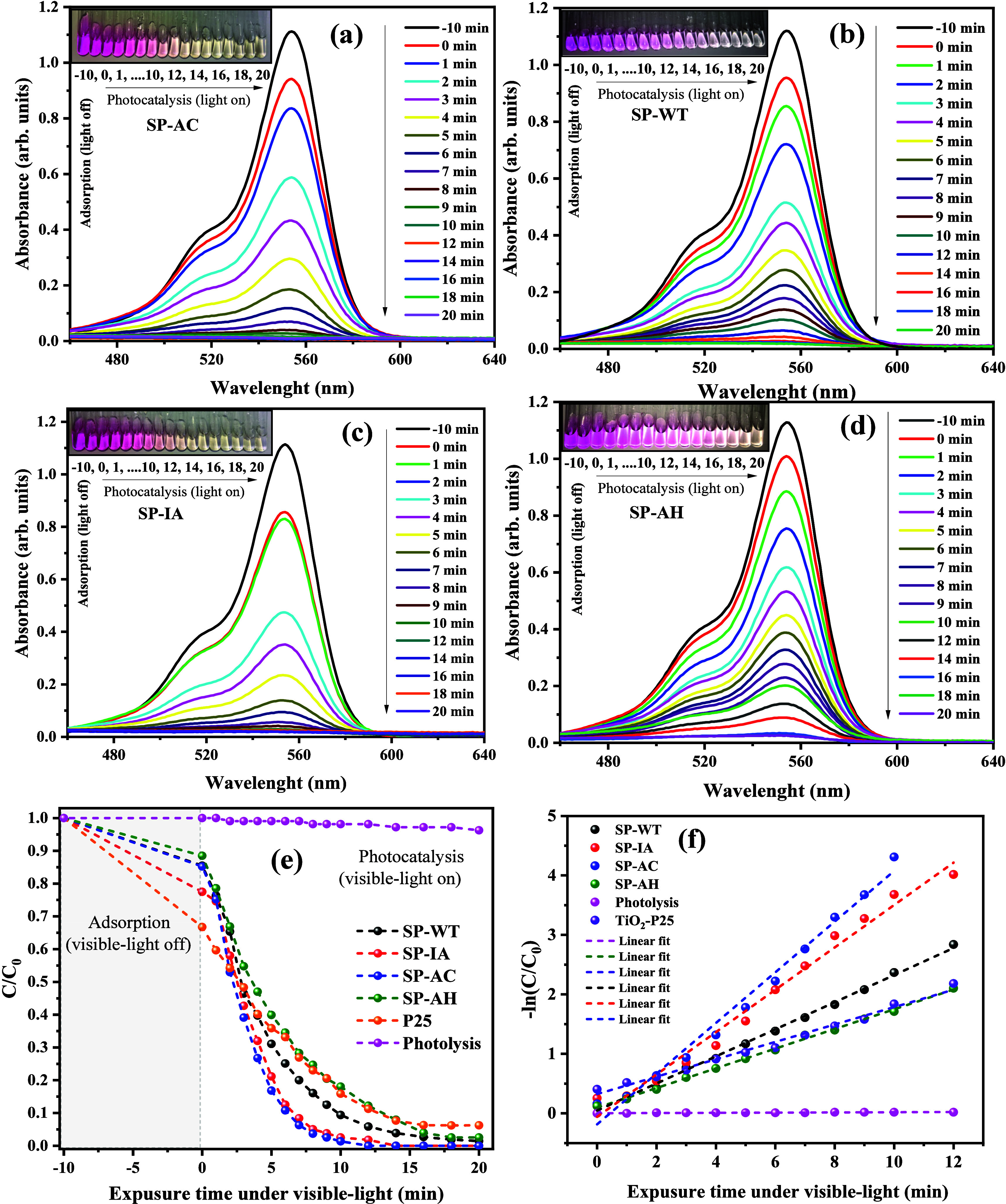
UV–vis spectrum for RhB dye against different exposure time in the presence of (a) SP-AC, (b) SP-WT, (c) SP-IA, and (d) SP-AH as photocatalyst and plot of (e) *C*/*C*_0_ and (f) −ln(*C*/*C*_0_) versus exposure time.

Figure 6(a–f) shows the UV–vis spectra in the region between 460 and 640 nm for the RhB dye, in the presence of the samples SP-AC, SP-WT, SP-IA, and SP-AH as photocatalysts, as well as the graphs obtained for the *C*/*C*_0_ and −ln(*C*/*C*_0_) against the time of exposure to visible radiation (LED) with a wavelength of 425 nm.

As seen in the images presented in Figure 6(a–d), it is possible to notice the evident decrease in the maximum absorbance associated with the wavelength at 554 nm, characteristic of the electronic transitions of the chromophore group of the RhB dye after the process of absorbing photons with a wavelength greater than 535 nm. This process includes, among other processes, N-deethylation, cleavage of the chromophore group, opening of the aromatic ring, and mineralization.^39,53,69−71^

Among the synthesized samples, it was confirmed that the SP-AC and SP-IA samples presented superior performance compared to the SP-WT and SP-AH samples. The percentage of degradation obtained after 20 min of exposure were: 100%, 98.2%, 94.2%, and 87.8%, referring to the samples SP-AC, SP-IA, SP-WT, and SP-AH, respectively. In addition, for photolysis (Figure S3, in supplementary electronic material), a percentage of 1.9% was obtained, while for TiO_2_, a percentage of degradation of 88.7% was obtained.

Figure 6(e–f) shows the plots for the kinetic profile of the photodegradation reaction are presented, in this case, adopting the pseudo-first-order kinetic model (eq 10),^72,73^ commonly adopted for photocatalytic processes using silver phosphate as a photocatalyst. In this respective equation, *C*_0_ and *C*_*t*_, correspond to the initial concentration and concentration at a given time (*t*), respectively. On the other hand, *k*_app_ is the apparent velocity constant of the reaction.
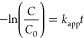
10The linear fit obtained for the -ln(C/C_0_) versus time of exposure to visible radiation, makes it possible to determine the apparent velocity constant of the reaction, which is equivalent to the angle coefficient of the line obtained for the adjustment of the points on the graph, as seen in Figure 6(f). The results obtained for the linear adjustment resulted in the values of *k*_app_ equals 426 × 10^–3^ min^–1^, 356.2 × 10^–3^ min^–1^, 227.8 × 10^–3^ min^–1^, 165.6 × 10^–3^ min^–1^, 156.2 × 10^–3^ min^–1^, and 1.63 × 10^–3^ min^–1^, corresponding to the samples SP-AC, SP-IA, SP-WT, SP-AH, TiO_2_-P25, and Photolysis, respectively. In all cases, the error in the determination of these values were less than 5%.

The half-life of the reactions was given by eq 11, using the condition that at a given time (*t*_1/2_), the final concentration (*C*) will be half of the initial concentration (*C*_0_), that is *C* = 0.5*C*_0_.
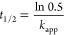
11Using the apparent velocity values calculated for the reactions performed in eq 10, it was possible to obtain the half-life time for the reactions as being equal to 0.2227 min, 0.3561 min, 0.4260 min, 0.1655 min, 1.636 × 10^–3^ min, and 0.1462 min, referring to the samples SP-WT, SP-IA, SP-AC, SP-AH, photolysis, and TiO_2_, respectively.

**Figure 7:**
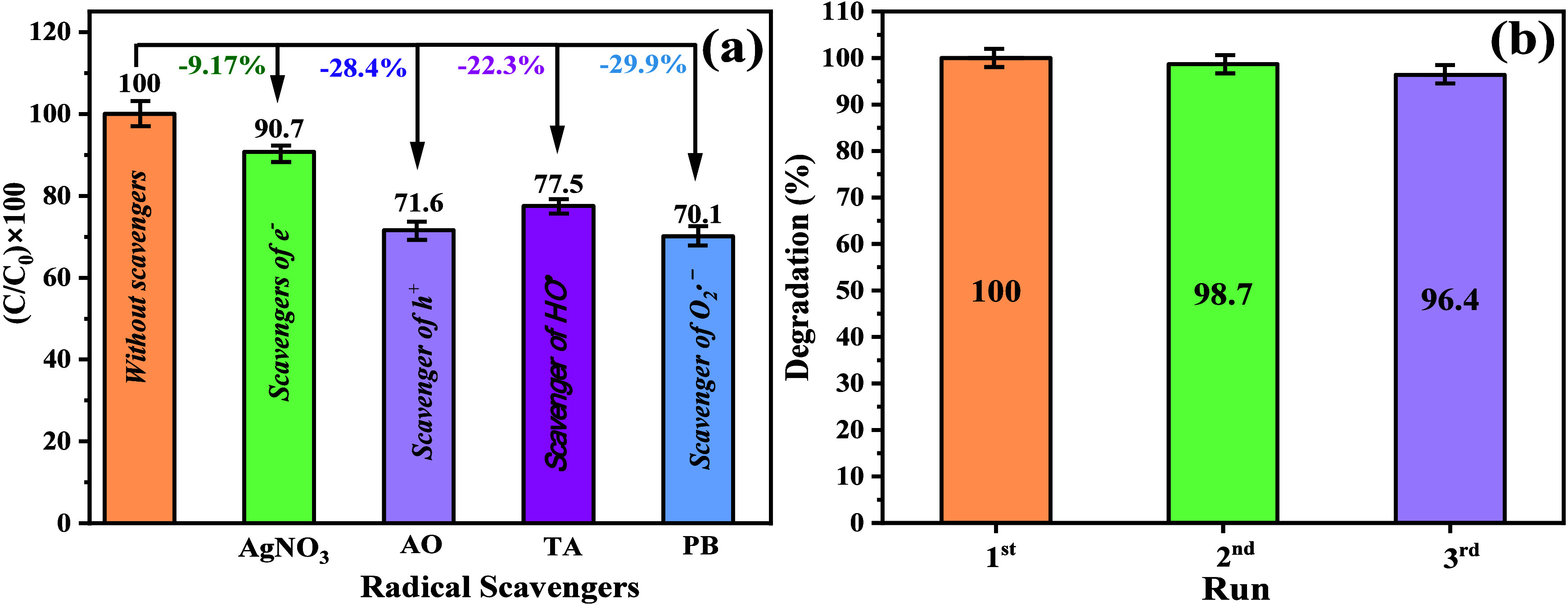
(a) Photocatalytic experiments with radical scavengers and (b) catalyst reusability around three consecutive cycles using the SP-AC sample as photocatalyst and RhB dye solution.

To investigate the contribution of each of the photogenerated species in the degradation of the RhB solution, a study was carried out with hydroxyl radial scavengers (HO^•^), superoxide radicals (O_2_^•–^), electrons (*e*^–^), and holes (*h*^+^), using the SP-AC sample as a catalyst. In this case, was decided to adopt the following scavengers compounds: terbutyl alcohol (TA), parabenoquinone (PB), silver nitrate (AgNO_3_) and ammonium oxalate (AO), respectively, as shown in Figure 7a.^74^ Based on these results, in the absence of the capturers, the performance of the SP-AC catalyst reached the percentage of 100% degradation of the RhB dye molecules after 20 min of visible radiation exposure. However, when the capturers AgNO_3_, AO, TA, and PB are adopted separately in the reaction environment, there was a reduction in photocatalytic performance by 9.17%, 28.4%, 22.3%, and 29.9%, respectively. Therefore, it is confirmed that the decreasing order of contribution of oxidative species to the processes involved in the degradation of RhB dye molecules is O_2_^•–^ > *h*^+^ > HO^•^ > *e*^–^. These results are in excellent agreement with the studies reported in the literature consulted.^6,25^

The stability of the catalyst Ag_3_PO_4_ (SP-AC), given the oxidative processes involved in the photodegradation of the RhB dye, was investigated through reuse in three consecutive cycles, as seen in Figure 7b. This study used 100 mg of the SP-AC sample in 100 mL of RhB dye solution at a concentration of 5 mg L^–1^, after 20 min exposure to visible LED radiation. As observed in the results presented in Figure 6b, the catalyst, even after the third catalytic cycle, showed a yield higher than 96%, indicating satisfactory stability against the oxidative processes involved in the photodegradation of the RhB dye molecules. In addition, the Figure S4 is shown the X-ray diffraction pattern collected of catalyst after the third cycle, where all diffraction peaks of characteristics of Ag_3_PO_4_ and Ag^0^, is confirmed, which the percentage of each phase are 79.7% and 20.3%, respectively.

In the study conducted by Tang et al.,^75^ silver phosphate microcrystals were obtained by chemical precipitation, obtaining different morphologies for the conditions adopted, when used in the photodegradation of methylene blue dye (MB), it was noted that in the third cycle of reuse, there was a reduction of approximately 15%. On the other hand, Zhang et al.,^29^ studied the attainment of heterojunction *n*-*p* between Ag_3_PO_4_ and cerium oxide (CeO), obtaining a reduction percentage of approximately 5.4% after three consecutive catalytic cycles. Therefore, based on the results obtained, the physicochemical properties of the SP-AC sample confirm a superior stability to the photochemical processes, which may be related to the short- and long-range structural organization in the crystal lattice, which induces the migration of electrons between the bands (VB and CB), under less effect of reduction of silver ions present in the structure.

The antimicrobial performance of microcrystals of Ag_3_PO_4_, was investigated against the strains of the bacteria *Staphylococcus aureus* (*S. aureus*), *Escherichia coli* (*E. coli*), *Pseudomonas aeruginosa* (*P. aeruginosa*), and fungus *Candida albicans* (*C. albicans*), using microdilution in 96-well plates. The initially investigated concentration of the biocide was 125 μg mL^–1^, which have been successively diluted to the concentrations of 62.5 μg mL^–1^, 31.25 μg mL^–1^, 15.63 mg mL^–1^, 7.81 μg mL^–1^, 3.90 μg mL^–1^, and 1.95 μg mL^–1^ as can be seen in Figure S5, available in supplementary electronic material. However, for the trials carried out with the control group, in this case, the positive control for the bacteria was the drug levofloxacin and for the strain of bacteria *S. aureus*, *E. coli*, and *P. aeruginosa*, The drug terbinafine was used, it was diluted from the same concentration but reached the concentration of 0.015 μg mL^–1^ for levofloxacin and 3.12 μg mL^–1^ for the strain of *C. albicans*.

There was significant antimicrobial activity for all samples investigated, confirming the effective potential of silver-based materials, especially silver phosphate, as a biocidal agent. Therefore, for the strains of *S. aureus*, *E. coli*, *P. aeruginosa*, and *C. albicans*, the lowest MIC values were 7.81 μg mL^–1^ (SP-IA and SP-AC), 7.81 μg mL^–1^ (SP-IA and SP-AC), 15.62 μg mL^–1^ (SP-IA, SP-AH, and SP-AC), and 7.81 μg mL^–1^ (SP-IA). On the other hand, the strains of *S. aureus*, *E. coli*, and *P. aeruginosa* exhibited MIC of 0.5 μg mL^–1^, 0.03 μg mL^–1^, and 0.48 μg mL^–1^ for the drug levofloxacin (positive control) and 6.25 μg mL^–1^ for the strain of *C. albicans* treated with the drug terbinafine. The negative control using distilled water shows that all cultivated strains grew normally, with no interference of the broth prepared for their nutrition, in the inhibiting growth. Figure S5, shows the photographic records of the plates used in the microbiological experiments to determine the MIC of the SP-AC, SP-IA, SP-WT, and SP-AH samples.

Based on the results presented in Table 2, it is possible to highlight that although the SP-AH sample presented antimicrobial activity, as well as photocatalytic activity in the assays against the RhB dye solutions, it exhibited the lowest effectiveness in the degradation processes of the RhB dye molecules, as well as the strains of the bacteria and fungus studied. Therefore, suggesting that the morphology of the microcrystals obtained, where the occurrence of microcrystals in polyhedral of heterogeneous dimensions and strong diffusion tendency between particles, does not present favorable characteristics to oxidative processes compared to the other samples. On the other hand, the sample synthesized using the mixture of acetone and distilled water solvents (SP-AC) showed high antimicrobial potential, as well as photocatalyst, which is directly related to the morphology of the microcrystals, which despite having different morphologies, the predominance of tetrapod-shaped microcrystals was confirmed. When compared with the studies reported in the literature (see Table 2), it is noted that the samples synthesized are among the most effective for the strains of tested microorganisms. Confirming, therefore, the high performance of the materials obtained by the proposed methodology.

**Table 2:** Antimicrobial Performance of SP-IA, SP-AH, SP-AC, and SP-WT against Different Microorganisms as Also Reported by Literature^21,76,20,23^^a^

	Minimum Inhibitory Concentration – MIC (μg mL^–1^)				
Sample	*S. aureus*	*E. coli*	*P. aeruginosa*	*C. albicans*	ref.
SP-IA	7.81	7.81	31.25	7.81	This work
SP-AH	31.25	15.62	31.25	15.62	This work
SP-AC	7.81	7.81	15.62	15.62	This work
SP-WT	15.62	15.62	31.25	31.25	This work
PC	0.5^#^	0.03^#^	0.48^#^	6.25*	This work
NC	NA	NA	NA	NA	This work
Ag_3_PO_4_	32	64	-	-	(21)
Ag_3_PO_4_	2000	500	-	2000	(76)
Ag_3_PO_4_	62.50	31.25	-	-	(20)
Ag_3_PO_4_	62.5	-	125	-	(23)

a The PC and NC are the positive and negative control respectively. Legend: SP-IA = Ag_3_PO_4_ synthesized with water/isopropyl alcohol as solvent; SP-AH = Ag_3_PO_4_ synthesized with water/ammonium hydroxide as solvent; SP-AC = Ag_3_PO_4_ synthesized with water/acetone as solvent; SP-WT = Ag_3_PO_4_ synthesized with water as solvent. # = Levofloxacin, * = Terbinafine, NC = negative control (bidistiled water), NA = no antimicrobial activity.

**Figure 8:**
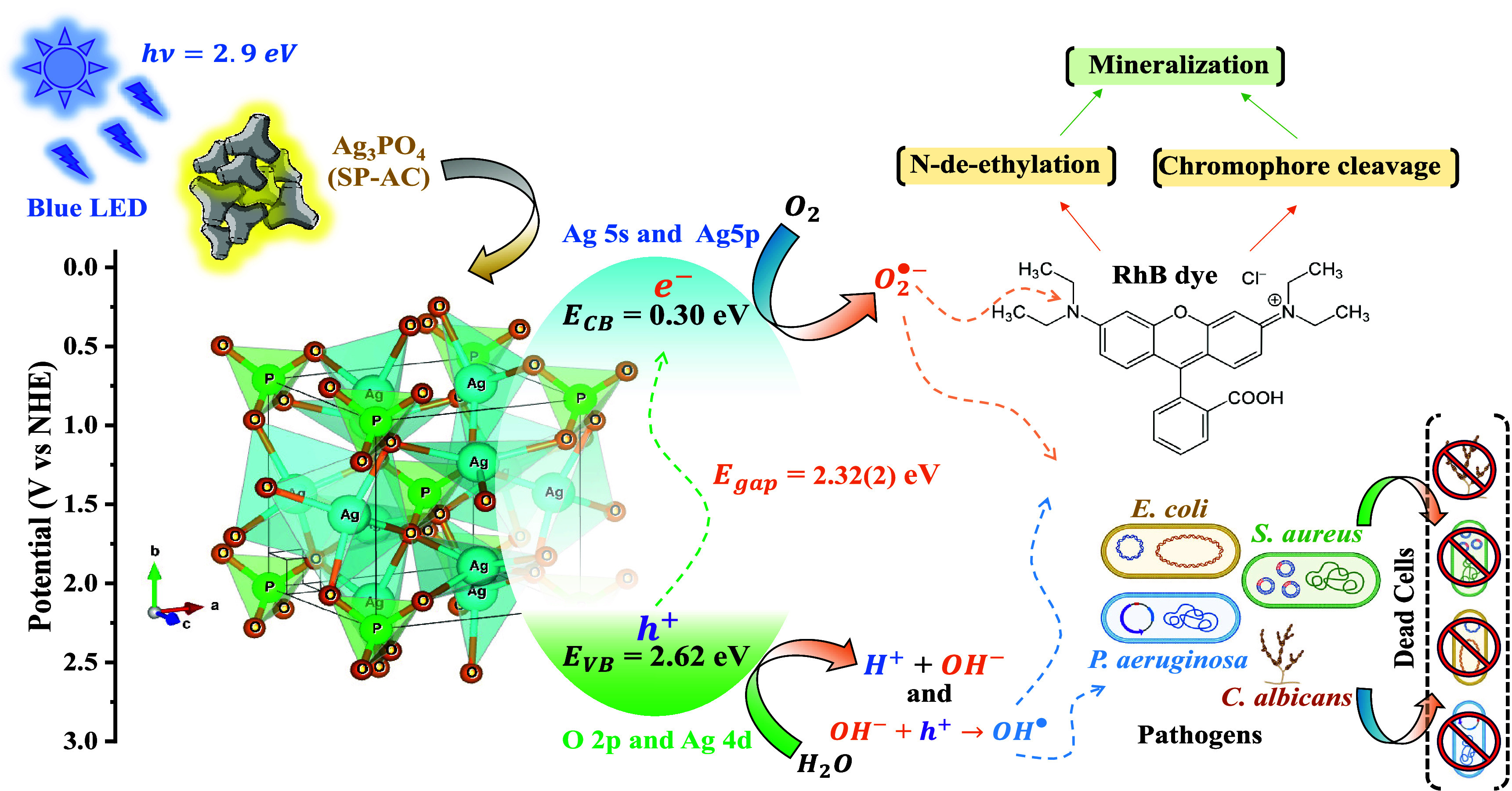
Schematic representation for photocatalytic and antimicrobial properties of Ag_3_PO_4_ (SP-AC) against RhB dye molecules and pathogens.

Based on the results obtained, we believe that the mechanism of photocatalytic activity, as well as antimicrobial activity, is related to the tetrapod morphology exhibited by the microcrystals contained mostly in the SP-AC sample, which have high exposure of the [110] direction, also reported by the literature as the most energetically favorable face to oxidative processes.^28^ Thus, when irradiated by visible light, the microcrystals undergo the process of excitation/recombination of electrons between the bands (CB and VB), since the energy from the LED light (2.9 eV) is higher than the value of the bandgap of the SP-AC sample. As presented in the discussion of the tests performed with the radical capturers and in the schematic representation in Figure 8, the greatest contribution to the degradation of the RhB dye molecules is associated with the superoxide radicals, hydroxyls, and holes, supporting the interpretations presented.

According to the literature,^77^ the photocatalytic efficiency of different materials is mainly associated with the recombination of the pair electron (*e*^–^)/hole (*h*^+^), provided by the absorption of light with a wavelength equal or greater than the bandgap of semiconductor. Where, the energy associated with the surface plane is fundamental in the mechanism of interaction with the molecules dissolved in the medium, as well as the transfer of charges throughout the structure, up to the surface of the microcrystals. In the present case, variations in atomic positions, differences in unit cell volume and variations in network parameters are strong indications of the presence of distorted [AgO_4_] and [PO_4_] clusters, which result in ordered structures ([AgO_4_]_o_ and [PO_4_]_o_) and disordered ([AgO_4_]_d_ and [PO_4_]_d_) along the crystal lattice, which is given by eqs 12 and 13.
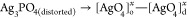
12
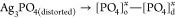
13When receiving blue photons, from LEDs with a wavelength of 420 nm (∼2.9 eV), electrons are excited to the conduction band, and holes are formed in the valence band, represented according to Kröger–Vink notation, adopting the symbols (′) and (•), for electron and hole, respectively. In this case, they result in the cluster [AgO_4_]_o_^′^–[AgO_4_]_d_^•^, as well as [PO_4_]_o_^′^–[PO_4_]_d_^•^, where the clusters [AgO_4_]_o_^′^ and [PO_4_]_o_^•^, are located in the conduction band, while the clusters [PO_4_]_d_^•^ and [PO_4_]_o_^•^ in the valence band. The described process is presented in eqs 14 and 15.
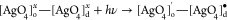
14
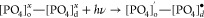
15In an aqueous medium, molecules of water (H_2_O) and oxygen gas (O_2_) are adsorbed on the surface of Ag_3_PO_4_ microcrystals, causing oxidation of water molecules by the clusters [PO_4_]_d_^•^ and [AgO_4_]_d_^•^, while oxygen molecules capture electrons from the [AgO_4_]_o_^′^ and [PO_4_]_o_^′^ clusters. In this process, the exposure of the surface planes, especially the {110} plane, favors the oxidation–reduction processes by the favorable energy of water and oxygen dissociation. In this study, was reported the high exposition of {110} with tetrapod morphology present for SP-AC sample. The oxidation of water molecules results in the formation of H^+^ and OH^–^ ions, as well as the oxidation of OH^–^ ions to hydroxyl radicals (HO^•^). On the other hand, oxygen molecules are reduced to superoxide radicals (O_2_^•–^). The reactions described are presented in eqs 16–21.
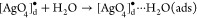
16
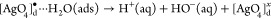
17
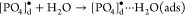
18
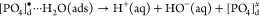
19As also,
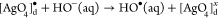
20
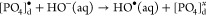
21The adsorption of the dye molecules on the surface of the microcrystals, with increased performance due to the {110} surface plane, also provides the opportunity for their direct oxidation, breaking the chemical bonds present in the molecule, into colorless byproducts of lower molecular weight (CCO), as shown in the following equations.
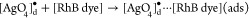
22
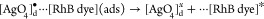
23
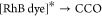
24
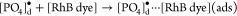
25
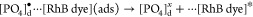
26
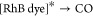
27In the conduction band, the occurs the following reactions.
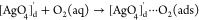
28
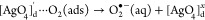
29
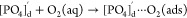
30
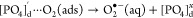
31Once generated in an aqueous medium, the radicals attack the carbon chains, mainly the bonds present in the chromophore group of the dye, destabilizing the structure and leading to the formation of colorless compounds of lower molecular weight (CCO), where occurs the N-dehethylation, cleavage and mineralization processes.
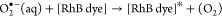
32
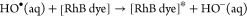
33
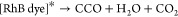
34Although the microbiological assays were not performed under the presence of light, based on the literature, it is possible to suggest that the high activity of silver phosphate microcrystals against the pathogens studied is directly related to the surface energy of the structures that, when subjected to the enzymatic processes promoted by the microorganisms, promote the formation of holes in the structures that provide the opportunity for the leaching of silver ions (Ag^+^).

Once in the middle, the ions Ag^+^ enter the cell interior through flow pumps and react with phospholipid chains present mainly in cytoplasmic organelles, causing the inactivation of basic cellular functions, including replication of genetic material and cellular respiration. On the other hand, the polarization of clusters [AgO_4_] and [PO_4_], leads to the formation of oxidizing species (HO^•^, O_2_^•–^, and *h*^*+*^) due to instability in aqueous media, they attack the bacterial wall, which is usually composed of peptidoglycans and phospholipids, causing damage that allows the release of intracellular fluid. Consequently, there is a weakening of cytoplasmic organelles and cell lysis. These results are in according to the reported by Singh et al.,^20^ which concluded that a high antimicrobial properties in the disruption of bacterial wall by silver phosphate microcrystals against Gram-positive and Gram-negative bacteria was archivied to microcrystals with a high exposure of {110} and {111} plan.

## Conclusion

Silver phosphate microcrystals with different morphologies were successfully obtained using the solvothermal method by adding different organic solvents in a 1:1 (v/v) water/solvent ratio. Therefore, the techniques employed in the materials characterization revealed that all synthesized samples exhibit pure phase with high crystallinity and vibrational modes characteristic of the cubic phase for silver phosphate. In addition, the morphological study revealed that the microcrystals synthesized with acetone/water, isopropyl alcohol/water, ammonium hydroxide/water, and water as a solvent exhibited tetraploids, rods, and irregular polyhedral morphologies, while when using only distilled water, it resulted in microcrystals with cube and polyhedron morphology. The optical bandgap calculated for the samples resulted in a value between 2.30 and 2.32 eV, which is characteristic of silver phosphate, which has strong absorption in the visible light region. In the photocatalytic assays against the RhB solutions, the SP-AC sample had better performance, associated with greater exposure of the {111} face, with the superoxide radicals and the holes that showed the greatest contribution to the oxidative processes. While in the microbiological assays, all samples exhibited biocidal activity, however, the SP-AC sample performed better than the others, for the strains of *E. coli* (7.81 μgmL^–1^), *E. aureus* (7.81 μgmL^–1^), *P. auruginosa* (15.62 μgmL^–1^), and *C. albicans* (15.62 μgmL^–1^). Therefore, it is a promising candidate for decontaminating effluents containing POPs and a material with high biocidal performance against pathogenic microorganisms.

## Supporting Information Available

Rietveld refinement results for atomic position, wyckoff, occupation; Williamson–Hall plot, schematic representation of unit cell; UV–vis diffuse reflectance spectroscopy plot (DRS) and reflectance (%) by colorimetry; UV–vis spectrum of RhB solution over photolysis experiment; and antimicrobial test by microdilution in 96-well cell culture plates (PDF)
