# Supplementary material for: Solvent Effect on the Structural, Optical, Morphology, and Antimicrobial Activity of Silver Phosphate Microcrystals by Conventional Hydrothermal Method
Source: ACS Omega. 2024 May 16;9(21):23069–85. doi: 10.1021/acsomega.4c02943 (PMC11137729; doi:10.1021/acsomega.4c02943)
Supplement: Supplementary file 1 — ao4c02943_si_001.pdf [file ao4c02943_si_001.pdf]

**Solvent effect on the structural, optical, morphology, and antimicrobial activity of silver phosphate microcrystals by conventional hydrothermal method**

Mitsuo Lopes Takeno[1#], Francisco Xavier Nobre[1*#], Fagner Ferreira da Costa[1], Marcus Valério Botelho do Nascimento[1], Wanison André Gil Pessoa Júnior[1], Edgar Alves Araújo Júnior[2], Giancarlo da Silva Sousa[2], Marcel Leiner de Sá[2], Raiana Silveira Gurgel[3], Patrícia Melchionna Albuquerque[3], José Milton Elias de Matos[2], Yurimiler Leyet Ruiz[4], and Carlos Roberto Grandini[5]

[1]*Department of Chemistry, Environment, and Food (DQA), Group of Energy Resources and Nanomaterials (GREEN), Federal Institute of Education, Science and Technology of Amazonas, Campus Manaus Centro, Manaus, 69020-120, AM, Brazil*

[2]*Interdisciplinar Laboratory of Advanced Materials-LIMAV, Federal University of Piauí-UFPI, Teresina, PI, Brazil.*

[3]*Research Group on Chemistry Applied to Technology, School of Technology, Amazonas State University, Manaus 69050-020, Brazil*

[4]*Department of Materials Engineering, Laboratory of Processing of Technological Materials (LPMaT), Federal University of Amazonas, Faculty of Technology, Rua Av. General Rodrigo Otávio Jordão Ramos, 1200, Coroado I, Manaus, 69067-005, Brazil*

[5]*Laboratório de Anelasticidade e Biomateriais, UNESP—Universidade Estadual Paulista, Bauru 17033-360, SP, Brazil*

[#]*These authors have equality contributed to this manuscript.*

**Corresponding author: Francisco X. Nobre (Francisco.nobre@ifam.edu.br)*

***Supplementary Electronic Material***

**Caption Table**

**Table S1:** Complementary Rietveld refinement results, such as lattice parameters, atomic position, unit cell volume (V) and occupation (Occ), for SP-AC, SP-WT, SP-IA and SP-AH samples, as also, the ICSD card nº. 14000.

| ♣atom | Atomic position | | | $O_{cc}$ | Lattice parameters (Å) | V (Å$^3$) |
|---|---|---|---|---|---|---|
| | x | y | z | | *a* = *b* = *c* | |
| **Ag** | 0.25000 | 0 | 0.50000 | 0.24552 | | |
| **P** | 0 | 0 | 0 | 0.09117 | 6.013(1) | 217.405(6) |
| **O** | 0.1326(3) | 0.1326(3) | 0.1326(3) | 0.21894 | | |

R profiles parameters: $R_{wp}$ = 22.5, $R_p$ = 30.2, $R_e$ = 15.78 and $\chi^2$ = 2.04.

| ♦atom | Atomic position | | | $O_{cc}$ | Lattice parameters (Å) | V (Å$^3$) |
|---|---|---|---|---|---|---|
| | x | y | z | | *a* = *b* = *c* | |
| **Ag** | 0.25000 | 0 | 0.50000 | 0.09035 | | |
| **P** | 0 | 0 | 0 | 0.25192 | 6.013(3) | 217.435(6) |
| **O** | 0.1476(3) | 0.1476(3) | 0.1476(3) | 0.33162 | | |

R profiles parameters: $R_{wp}$ = 22.5, $R_p$ = 26.1, $R_e$ = 18.02 and $\chi^2$ = 1.56.

| ♥atom | Atomic position | | | $O_{cc}$ | Lattice parameters (Å) | V (Å$^3$) |
|---|---|---|---|---|---|---|
| | x | y | z | | *a* = *b* = *c* | |
| **Ag** | 0.25000 | 0 | 0.50000 | 0.12964 | | |
| **P** | 0 | 0 | 0 | 0.04821 | 6.013(1) | 217.408(3) |
| **O** | 0.1529(4) | 0.1529(4) | 0.1529(4) | 0.16922 | | |

R profiles parameters: $R_{wp}$ =17.6, $R_p$ = 23.9, $R_e$ = 18.18 and $\chi^2$ = 0.936.

| ♠atom | Atomic position | | | $O_{cc}$ | Lattice parameters (Å) | V (Å$^3$) |
|---|---|---|---|---|---|---|
| | x | y | z | | *a* = *b* = *c* | |
| **Ag** | 0.25000 | 0 | 0.50000 | 0.12096 | | |
| **P** | 0 | 0 | 0 | 0.04312 | 6.013(1) | 217.406(4) |
| **O** | 0.1499(1) | 0.1499(1) | 0.1499(1) | 0.15442 | | |

R profiles parameters: $R_{wp}$ = 18.2, $R_p$ = 19.7, $R_e$ = 14.38 and $\chi^2$ = 1.60.

| Ψatom | Atomic position | | | $O_{cc}$ | Lattice parameters (Å) | V (Å$^3$) |
|---|---|---|---|---|---|---|
| | x | y | z | | *a* = *b* = *c* | |
| **Ag** | 0.25000 | 0 | 0.50000 | 0.25000 | | |
| **P** | 0 | 0 | 0 | 0.08333 | 6.0260 | 218.820 |
| **O** | 0.1476(3) | 0.1476(3) | 0.1476(3) | 0.33333 | | |

**Legend:** ♣ = SP-AC; ♦ = SP-WT; ♥ = SP-IA; ♠ = SP-AH; Ψ = ICSD card nº. 14000.

**Caption Figure**

Figure S1 and S2 show the reflectance spectra of samples SP-AC, SP-WT, SP-IA and SP-AH, where increasing reflectance values are observed for wavelengths related to energy values equal to or greater than 2.3 eV. In contrast, for values below 2.3 eV, a significant reduction in reflectance values is noted, due to the strong absorption of photons by silver phosphate microcrystals, in the visible region.

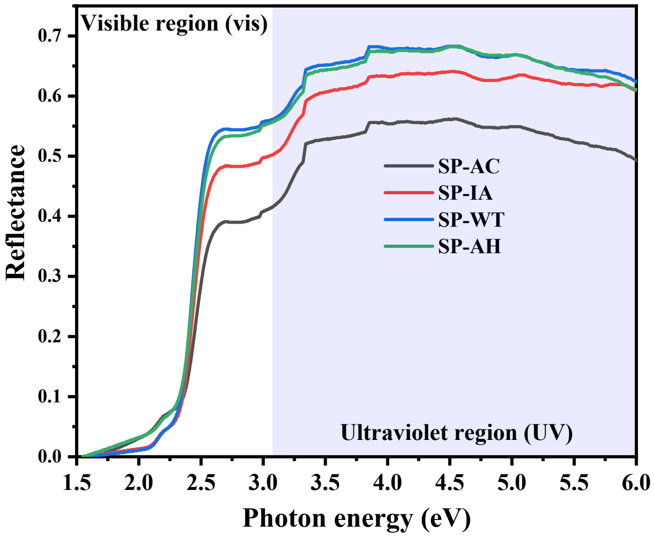


**Figure S1:** UV-Vis by diffuse reflectance spectroscopy of samples.

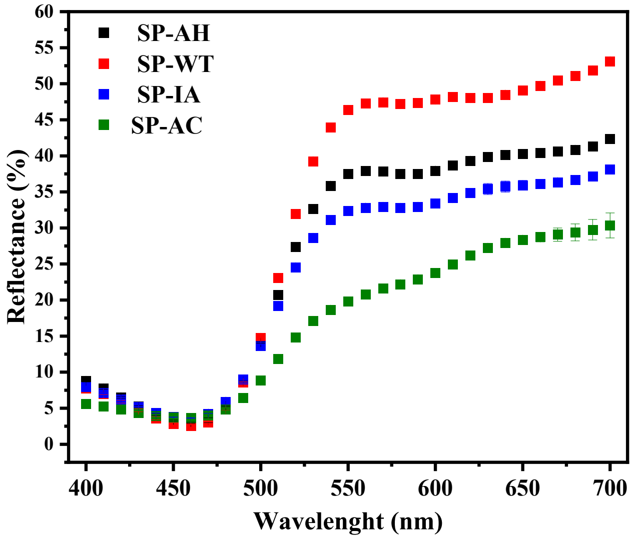


**Figure S2:** Diffuse reflectance spectroscopy by colorimetry of samples.

The UV-vis spectrum of the RhB dye in solution is shown in Figure S3, for different times of exposure to UV-LED visible radiation, referring to the photolysis tests. In this case, it is possible to notice the inefficiency in the photodegradation of the RhB dye molecules by the action of visible light, confirming the need to add

high-performance catalysts with properties that satisfy the oxidative processes and consequently degrade the dye molecules, as verified for the synthesized catalysts SP-AC, SP-WT, SP-IA and SP-AH.

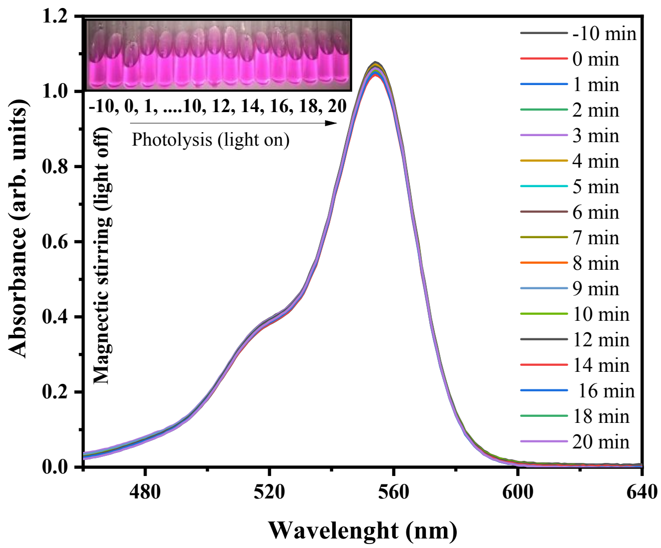


**Figure S3:** UV-vis spectrum of RhB dye solution of photolysis experiment.

Os padrões de difração da amostra SP-AC antes e posterior a três ciclos fotocatalíticos frente ao corante RhB, estão apresentados na Figura S4, onde é possível notar a formação de prata metálica mediante os processos oxidativos, confirmados pelos picos de difração na posição 2θ em 38.3º, 44.3º e 64.59º, associados aos planos cristalográficos (111), (200) e (200), respetivamente. A quantificação do percentual de Agº na composição da amostra foi realizado integrando a área correspondente aos picos de maior intensidade para o $Ag_3PO_4$ e Agº, portanto, os planos associados aos picos em 2θ = 33.2º (210) e 38.3º (111), respectivamente. Posteriormente, foi utilizado a relação entre as áreas apresentado na Equação S1.

The diffraction patterns of the SP-AC sample before and after three photocatalytic cycles against the RhB dye are shown in Figure S4, where it is possible to notice the formation of metallic silver through oxidative processes, confirmed by the diffraction peaks at position 2θ at 38.3º, 44.3º and 64.59º, associated with the crystallographic planes (111), (200) and (200), respectively. The quantification of the percentage of Agº in the sample composition was carried out by integrating the area corresponding to the highest intensity peaks for Ag3PO4 and Agº, therefore, the planes associated with the peaks at 2θ = 33.2º (210) and 38.3º (111), respectively. Subsequently, the relationship between areas presented in Equation S1 was used.

$$\%Ag^{o} = \frac{A_{(111)}}{A_{(210)} + A_{(111)}} \times 100 \qquad (S1)$$

Where, $A_{(111)}$ and $A_{(210)}$ correspond to the area of the peaks associated with the crystallographic planes (210) and (111) associated with the structures of silver phosphate and metallic silver, respectively. Therefore, obtaining the percentages of 20.3% of $Ag^{o}$ and 79.7% of $Ag_3PO_4$, respectively.

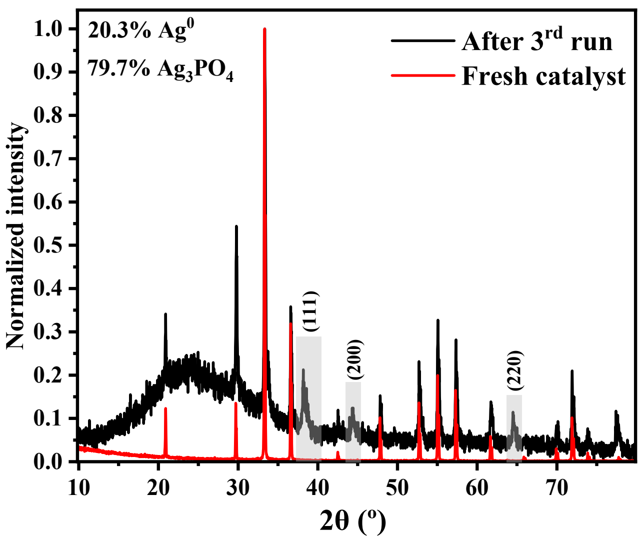


**Figure S4:** XRD diffraction pattern of SP-AC samples before and after three consecutive photocatalytic cycles.

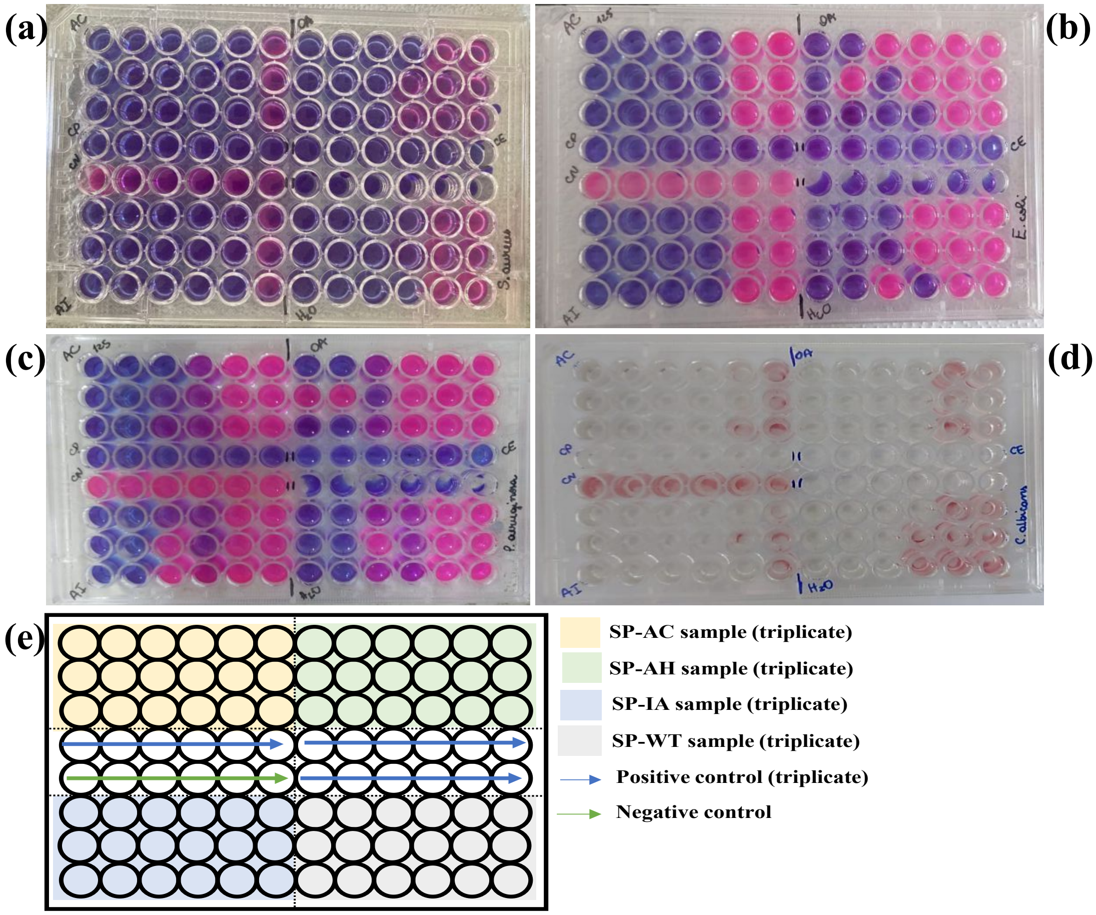


**Figure S5:** Antimicrobial assay to obtain the Minimum inhibitory Concentration (MIC) against (a) *Staphylococcus aureus*, (b) *Escherichia coli* (c) *Pseudomonas aeruginosa* and (d) *Candida albicans*. (e) schematic representation for tested samples and controls in the 96-well plate used in the experiments.

## Crystallographic information's of synthesized samples

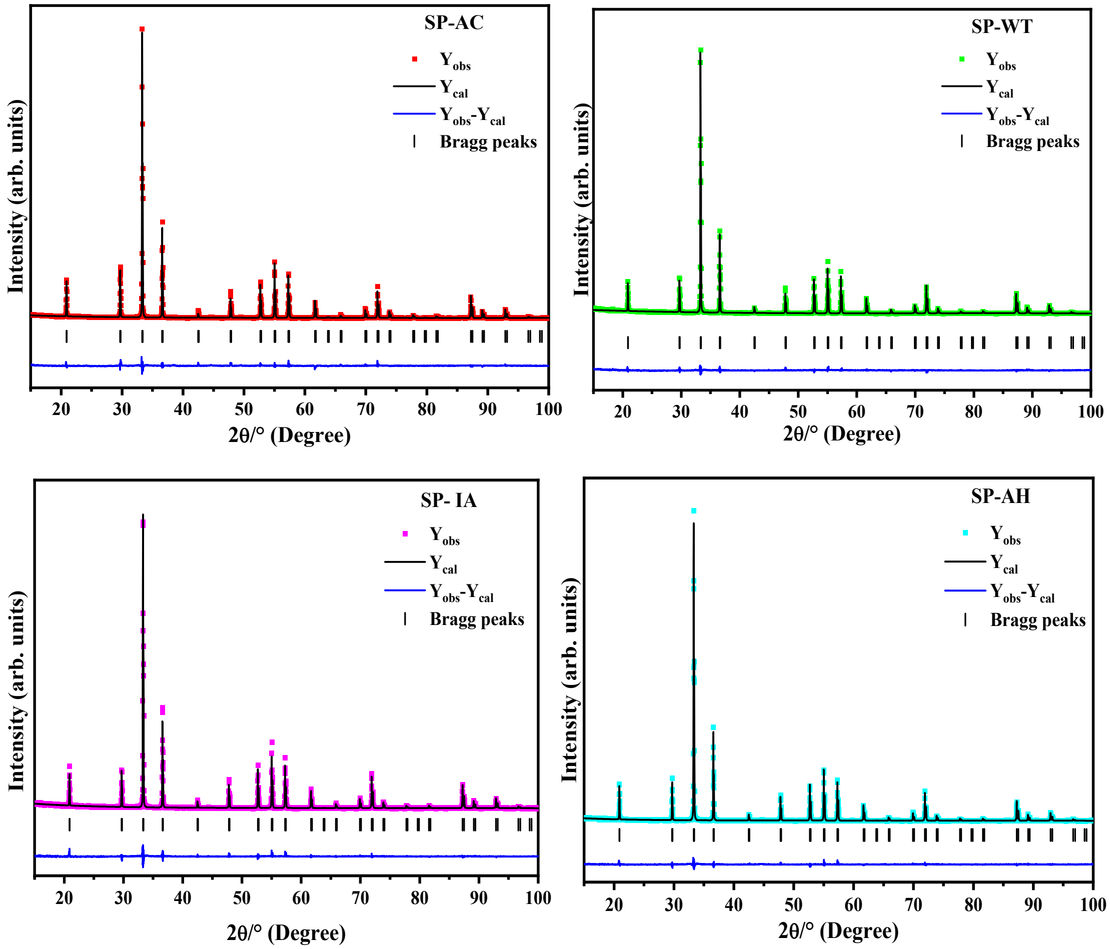


**Figure S6:** Structural Rietveld refinement plot of SP-AC, AP-WT, SP-IA and SP-AH samples.

**Table S2:** Crystallographic data from structural Rietveld refinement for $Ag_3PO_4$ samples denoted as SP-AC, AP-WT, SP-IA and SP-AH.

| **Crystallographic data** | **Samples** | | | |
|---|---|---|---|---|
| | SP-AC | SP-WT | SP-AH | SP-IA |
| Source | X-ray | X-ray | X-ray | X-ray |
| Chemical formula | $Ag_3PO_4$ | $Ag_3PO_4$ | $Ag_3PO_4$ | $Ag_3PO_4$ |
| Formula weight | 418.58 | 418.58 | 418.58 | 418.58 |
| Temperature | room temperature | room temperature | room temperature | room temperature |
| Pressure | ambient | ambient | ambient | ambient |
| Wavelength (nm) | CuKα = 0.154056 | CuKα = 0.154056 | CuKα = 0.154056 | CuKα = 0.154056 |
| Crystals system | cubic | cubic | cubic | cubic |
| Space group | $\overline{P}43n$ | $\overline{P}43n$ | $\overline{P}43n$ | $\overline{P}43n$ |
| a=b=c (Å) | 6.013(1) | 6.013(3) | 6.013(1) | 6.013(1) |
| α = β = γ (°) | 90 | 90 | 90 | 90 |
| V (Å$^3$) | 217.405(6) | 217.435(6) | 217.406(4) | 217.408(3) |
| Z | 8 | 8 | 8 | 8 |
| d-space range (2θ) | 10°-100° | 10°-100° | 10°-100° | 10°-100° |
| $\chi^2$ | 2.04 | 1.46 | 1.60 | 2.11 |
| $R_{exp}$ | 15.8 | 17.6 | 14.4 | 8.70 |
| $R_{wp}$ | 22.5 | 21.2 | 18.2 | 12.6 |

**Legend:** $R_{wp}$ = weighted profile; $R_{exp}$ = expected.
